# Healthcare utilization and costs of early childhood mental health problems: a longitudinal analysis using multi-rater assessments and Swedish national registers

**DOI:** 10.1186/s13034-026-01110-8

**Published:** 2026-06-12

**Authors:** Ingyin Moe, Natalie Durbeej, Claire de Oliveira, Filipa Sampaio

**Affiliations:** 1 Uppsala Health Economics, Department of Public Health and Caring Sciences, Uppsala University, Uppsala, Sweden; 2 Department of Health Sciences, Karlstad University, Karlstad, Sweden; 3 Campbell Family Mental Health Research Institute, Institute for Mental Health Policy Research, Centre for Addiction and Mental Health, Toronto, Canada; 4 Institute of Health Policy, Management and Evaluation, Dalla Lana School of Public Health, University of Toronto, Toronto, Canada; 5 Department of Learning, Informatics, Management and Ethics, Karolinska Institutet, Stockholm, Sweden

**Keywords:** Preschool children, Early mental health problems, Healthcare utilization, Costs, Economic impact, Sweden

## Abstract

**Background:**

Mental health problems in preschool children are rising in Sweden, yet their economic impact on the health sector remains poorly quantified. This study estimated the incremental healthcare utilization and costs associated with early mental health problems to inform resource allocation and early intervention.

**Methods:**

We analyzed an exact matched sample of 1206 drawn from a cohort of 6957 children aged 3–5 from Uppsala Region, Sweden. Early mental health problems were identified using the Strengths and Difficulties Questionnaire reported by mothers, fathers and teachers. Data were linked to national registers to estimate cumulative healthcare utilization and costs over four years from a health sector perspective. Hurdle models, generalized linear models, and two-part models compared utilization and costs between children with and without early symptoms. Sub-group analyses by sex, symptoms, and raters were performed.

**Results:**

Children with early mental health problems consumed three additional units of medication with an incremental cost of US$ 299, one additional specialized outpatient visit with an incremental cost of US$ 622, and five additional primary care visits with an incremental cost of US$ 376 compared to their healthy peers. Healthcare utilization and costs were higher for boys with externalizing and internalizing symptoms. Findings were consistent across all rater groups.

**Conclusions:**

Early childhood mental health problems represent a substantial economic impact to the healthcare sector. The findings suggest that early identification and intervention in the preschool years may offer significant opportunities for cost-offsetting and improved long-term system efficiency.

**Supplementary Information:**

The online version contains supplementary material available at10.1186/s13034-026-01110-8.

## Key points for decision makers

- Early childhood mental health problems are associated with higher costs and utilization across primary, specialized, and pharmaceutical care within just four years of identification.
- Such impacts are observable as early as age 3, suggesting that the “window of opportunity” for cost-saving interventions begins in the preschool period.
- High consistency across parent and teacher ratings suggests that multi-informant screening is a robust tool for identifying children likely to drive high healthcare utilization.

**Supplementary Information:**

The online version contains supplementary material available at10.1186/s13034-026-01110-8.

## Introduction

Mental health problems among children and adolescents stand out as a pivotal area where a heightened disease burden is evident, constituting to a global prevalence of 13% [1]. According to a meta-analysis conducted by Vasileva et al. [2], children aged 1–7 years are reported to have a high prevalence of mental disorders, indicating the particular need of early targeted interventions among this age group. Children experiencing poor mental health and emotional difficulties often face a range of adverse outcomes, including poor academic performance, higher rates of school absenteeism, social difficulties, substance use, and increased risk of physical health problems [3–6]. According to a national estimate of the economic impact of child mental disorders in the UK, these conditions were associated with additional health, social care and education costs, totaling £1.4 billion in 2008 [7]. Likewise, research from other countries has reported that children with mental health problems have large healthcare utilization and related expenditures [8–13], and continue to exhibit high mental healthcare service demands and related costs in adulthood [14, 15]. Moreover, mental health problems not only affect children themselves, but also their families. These problems can lead to broader societal repercussions, such as increased utilization of social and healthcare services and lost productivity among caregivers, compared to their healthy counterparts [16].

In Sweden, mental health problems among children and adolescents have increased notably in recent decades, manifesting in both psychological and somatic complaints [17, 18]. In 2021, around 6% of the population aged 0–17 years had been in contact with child and adolescent psychiatry, with about one third of them seeing a healthcare professional for Attention Deficit Hyperactivity Disorder (ADHD) [19]. With increasing mental health problems in children, higher service utilization and costs have also been reported. A study from Sweden by Sampaio et al. [20] using data from the Children and Parents in Focus Study reported that preschool children with both somatic and mental health problems had higher service use, particularly at school and at home (such as speech and language therapy, child and social welfare services) than children experiencing no problems, with mean annual costs of US$ 13,826 and US$ 1583, respectively. However, this study used a cross-sectional design and included only reports from one primary caregiver [20]. Another study exploring the healthcare utilization between 2016 and 2018 in children and adolescents with psychiatric disorders in the Västra Götaland region in Sweden observed that children aged 3–17 years old with psychiatric diagnoses had more primary care visits, somatic outpatient and inpatient care visits [21]. However, the authors did not estimate costs associated with healthcare utilization.

Most mental health problems have an onset between childhood and early adulthood, highlighting the importance of access and early intervention after first presentation of illness [22]. Despite the vast literature on healthcare utilization and expenditure associated with mental health problems in children and adolescents, data on preschool children is scarce, particularly in the Swedish context [23]. According to Dave et al. [24], fathers and mothers tend to provide different ratings of their children’s mental health problems. Thus, using only one rater could lead to either under- or overestimation of such problems. To account for these gaps in knowledge, we aimed to estimate the cumulative incremental healthcare utilization and costs associated with early mental health problems in preschool children, over a 4-year time horizon, using data from a Swedish longitudinal observational study. Our study also considered reports of early mental health problems from multiple raters (mothers, fathers and preschool teachers), and examined differences by sex and type of mental health problems.

## Methods

### Study design

This longitudinal register-based study used cohort data from the “Children and Parents in Focus trial,” collected between 2013 and 2017 in Uppsala Region, Sweden [25]. The cohort data was linked to longitudinal data on inpatient, specialized outpatient and primary care, as well as prescribed medication from Swedish national registers from the date of cohort inclusion (2013–2017) until 2021.

### Participants and procedure

This study included 6957 unique children aged 3–5 years old who were recruited via Child Health Centers (CHC) in Uppsala Region prior to their annual health check-ups and whose parents/guardians consented to participate in the study during the period of 2013–2017. Prior to the yearly check-up, CHC nurses sent out a reminder and a consent form to the child´s primary caregiver along with three sets of questionnaires—one for each parent and one for preschool teachers. All parents of 3-, 4- and 5-year-old children who attended the check-ups could participate in the study. The questionnaires were available in Swedish, English, Arabic, Persian, Sorani and Somali. Parents who did not understand these languages were excluded. The questionnaires included the Strengths and Difficulties Questionnaire (SDQ) to measure children’s mental health problems [26], the 12-item General Health Questionnaire (GHQ-12) to assess parental psychological distress [27, 28], as well as questions on sociodemographic background of children and their parents.

### Exposure variable (early mental health problems)

Early mental health problems were assessed at ages 3, 4 and 5 years using the SDQ, a 25-item validated questionnaire with 5 subscales: emotional symptoms, conduct problems, hyperactivity/inattention, peer relationship problems, and prosocial behavior [26]. Total difficulties scores were generated by adding the scores of the first four sub-scales. Published norms derived from a Swedish sample of 3–5 year-olds were used to establish clinical cut-offs for mental health problems [29]. Based on the total difficulties score, children were classified into two groups. Children scoring above the pre-defined age- and sex-specific cut-offs on the total difficulties score were categorized as children with early mental health problems, while those scoring below the cut-offs were classified as children without early mental health problems. In addition, internalizing symptom scores were derived by summing the emotional and peer problem subscale scores, while conduct problem and hyperactivity subscale scores were combined to produce externalizing symptom scores.

Mothers’ ratings on SDQ were used as base-case analysis as they were the most prevalent raters in the cohort (mother raters, *n* = 6720 compared to father raters, *n* = 6591) and given the differences in ratings of child behavior noted in previous research [24, 30]. As some children were assessed more than once, the earliest SDQ assessment was chosen to identify mental health problems in the children, ensuring the capture of earliest signs. This same approach was taken in previous work [31] to (1) assess child mental health problems as early as possible; (2) extend the follow up time; and (3) capture health services for children with mental health problems early on.

### Outcomes

Outcomes included cumulative healthcare utilization and related costs estimated from the earliest SDQ assessment until ages 7–9. Costs were estimated from the Swedish health sector perspective, including direct healthcare costs and patients’ out-of-pocket expenses.

#### Healthcare utilization and costs

Inpatient and specialized outpatient care data were sourced from the National Patient Register (NPR) held by the Swedish National Board of Health and Welfare (Socialstyrelsen) [32]. The NPR provided data on the main and secondary diagnoses classified according to International Classification of Disease (ICD) codes (ICD version 10) as well as information on dates, types, frequencies, and duration of services used. Coverage of the NPR is approximately 100% for inpatient and 80% for outpatient services. For each of the inpatient stay and specialized outpatient visit, a specific Nordic Diagnostic Related Group (NordDRG) code was used. Costs related to inpatient and specialized outpatient care resource were estimated based on yearly DRG weights available from the Socialstyrelsen [33]. Each DRG weight was assigned a unit cost value for each year between 2013 and 2021. For this period, the estimated yearly monetary value for a DRG unit was between 42,265 SEK (Swedish Kronor) and 66,156 SEK.

$$\begin{aligned} DRG\;cost\;estimate & = DRG\;weight \times \\ & \quad cost\;per\;DRG\;unit\;for\;a\;given\;year \end{aligned}$$

Prescribed medication data were sourced from the Prescribed Drug Register (PDR) and included variables on prescription date, dispensation date, medication dosage and number of packages, drug type (generic/original/imported), and different cost categories (total, additional, cost per package, out-of-pocket costs) in SEK. Drug costs were sourced from the Swedish PDR using market prices. Furthermore, to assess whether the presence of early mental health problems was correlated with the use of specific drugs, relevant Anatomical Therapeutic Chemical Classification System (ATC) codes were identified based on available data. Given that only 3-digit ATC codes were available in the data, the following were considered: neuroleptics (N05), psycho-analeptics (N06) and other nervous system drugs (N07). In our analysis, total costs of medication were considered.

Primary care data were obtained from the Region Uppsala´s administrative system and included information on types of primary care visit (e.g., home visit, health center visit, telephone call, emergency visit), date of visits, main and sub diagnoses, and names of primary care centers. Relevant costs were estimated based on mean unit costs sourced from the Swedish cost per patient database [34]. Total primary care costs were calculated by summing the unit costs of each visit type for each individual.

Total cumulative utilization and associated costs over the 4-year follow-up were calculated for each child in each group (with early mental health problems vs. without), as the product of resource units by relevant unit costs. Because the dataset only included individuals with complete follow-up data, there was no attrition, and all cumulative estimates reflect full observation periods. Healthcare costs were inflated to 2024 Swedish Krona (SEK) based on consumer price index [35] and subsequently converted to 2024 U.S. dollars (US$) using Purchasing Power Parities for Gross Domestic Product [36].

### Statistical analyses

#### Base case analysis

Baseline characteristics, and average utilization and costs for each group (children with and without early mental health problems) were analyzed descriptively. Incremental cumulative healthcare utilization and associated costs were estimated using appropriate regression models, defined as the differences between children with early mental health problems and those without.

As part of the descriptive analyses, we also identified whether each child with and without early mental health problems had received any formal psychiatric diagnosis or medication during the follow-up period using ICD-10 codes F00–F99 (mental and behavioral disorders) from the NPR, and ATC codes of N05, N06, and N07 from the PDR.

#### Covariates

Informed by Andersen’s Behavioral Model, a set of observed individual-level variables were selected as model covariates to account for factors that may influence access to healthcare services [37]: child age and sex, parents’ education level, parents’ country of birth, parents’ marital status, number of children in the household, and parental psychological distress. Several variables were recoded: parental marital status (originally five categories) was recoded into two (married/cohabiting; and other); parental education (five categories) into three (primary school; high school; and university); and number of children in the household into three categories (1 child; 2 children; and ≥ 3 children). Parental psychological distress was measured using the 12-item General Health Questionnaire (GHQ-12), a validated self-report screening tool including symptoms of anxiety, depression, social dysfunction, and loss of confidence [28]. Higher scores reflect greater distress. Based on established scoring thresholds [28], a GHQ-12 score of ≥ 12 was used to classify parents into a group with psychological distress and < 12 into a group without. Variable recoding is detailed in supplementary material Table 1.

#### Matching of participants

To ensure comparability between groups, children with early mental health problems were exact matched with children without early mental health problems on relevant confounders: sex (males, females), age (3, 4, 5 years old), parental education (primary school, high school, university), country of birth (Sweden, other), and parental psychological distress (yes, no). Parental marital status and number of children in the household were excluded from the matching due to their limited predictive value for healthcare utlization and costs [30], but were retained as covariates in subsequent regression models to adjust for potential confounding. Exact matching was conducted in R using the *MatchIt* package, employing nearest neighbor matching without replacement [38]. Covariate balance between groups before and after matching was assessed using the standardized mean difference (SMD), with SMD = 0 indicating no difference between the groups. Descriptive statistics were used to outline sample characteristics and assess the sample balance, both pre- and post-matching.

#### Analysis of utilization and cost data

Cumulative healthcare utilization and costs were presented as descriptive statistics for each health service category as well as for total health service. Between-group differences were calculated as the difference in group averages with corresponding standard errors and 95% confidence intervals. To estimate the change in healthcare utilization and costs over time, average healthcare utilization and costs were estimated yearly for each group (see detailed results on yearly average cumulative utilization and costs in supplementary material Figs. 1 and 2).

Utilization data and healthcare costs typically display distinct statistical characteristics: they are often non-negative (i.e., they take on continuous values greater than zero), substantially right skewed with long tails, and frequently included a large portion of zeros (indicating null consumption or cost). Using simple linear regression models such as ordinary least squares (OLS) to estimate such outcomes would result in mis-specified models with biased estimates as they assume normal distribution and constant variance [39]. Thus, a comprehensive assessment of model assumptions and data distribution was conducted prior to the regression analysis. Each utilization and cost variable was first evaluated for overdispersion using the Pearson χ²/df ratio, with values greater than one indicating overdispersion, signifying that the variance exceeds the mean and that standard Poisson or linear models may be inadequate due to unaccounted heterogeneity or clustering in the data. The test for normality was evaluated using the skewness-kurtosis test and visualized with quantile–quantile (Q–Q) plots. Homoskedasticity was tested using the Breusch-Pagan test [40], with low p-values suggesting the presence of heteroskedasticity. The appropriate link and distribution family of utilization and cost data were chosen based on the results of the modified Park test [41]. Most importantly, the proportion of zeros were calculated for each outcome variable, with > 10% suggesting a large zero mass based on a common rule of thumb [42]. Model goodness-of-fit was further guided by the Akaike Information Criterion (AIC) and Bayesian Information Criterion (BIC) estimates. Details on model diagnostics can be found in supplementary material Table 2.

Based on the diagnostic results—the modified Park test, high proportion of zeros, substantial right-skewness of the distribution, heteroskedasticity, and strong overdispersion of count and continuous data—a Hurdle model with negative binomial distribution for healthcare use and a two-part model with gamma distribution for costs were used to model the difference in cumulative healthcare consumption and costs between children with and without mental health problems for each healthcare category [39]. Both the hurdle and two-part models were composed of two distinct parts. In the hurdle model, the first part employed a logit model to estimate the probability of having zero visits, and the second part used a truncated negative binomial model to model the count of visits among those with non-zeros. Similarly, in the two-part models, a logit model was employed to predict the probability of zero expenditure, followed by a generalized linear model (GLM) with a gamma distribution and log link to model the positive expenditure values. Both first and second components of the models were jointly interpreted as a whole model output. Since the model coefficients were not directly interpretable, marginal effects were estimated instead. Regarding the total healthcare consumption and costs, a GLM with negative binomial distribution for count data, and a GLM with gamma distribution for costs were selected due to low zero mass (< 10%) [43].

#### Sub-group analyses

Two sub-group analyses were conducted. First, average cumulative utilization and costs among children with mental health problems was descriptively analyzed by sex for each healthcare category. Differences in mean values between the groups were reported with corresponding 95% CI.

Using SDQ subscale cut-off norms from a working paper, children with early mental health problems were categorized into three symptom-based groups: those exhibiting only internalizing symptoms, only externalizing symptoms, and those with both (comorbid). For each symptom group, average healthcare utilization and associated costs were calculated across all types of health services. To investigate potential sex differences within these mental health profiles, each of the three groups was further stratified by sex. This allowed for a comparison of healthcare use and costs between boys and girls within each symptom category.

#### Sensitivity analyses

Sensitivity analyses were conducted using SDQ ratings reported by fathers and preschool teachers. Similar analytical approach was used as in the base case analysis. However, for the analysis of preschool teachers’ rating, exact matching was limited to sex and age of children due to the absence of parental socio-demographic information in the teachers’ questionnaire.

Matching was done in R. All other analyses were performed using StataNow/MP 18.5.

## Results

### Base case

**Fig. 1:**
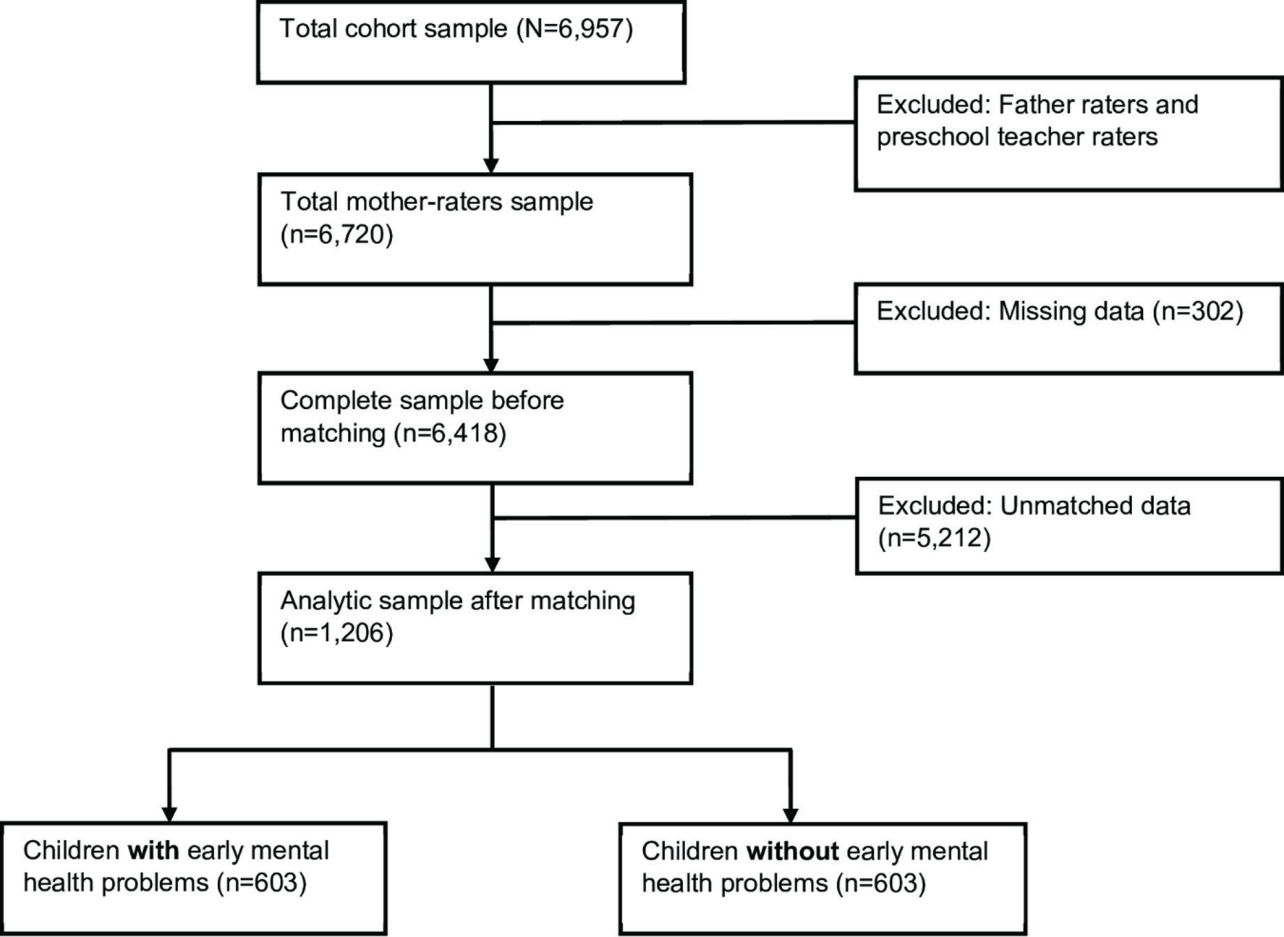
Study population flowchart (Mother-rated SDQ)

The initial base case mother-rater sample (*n* = 6720) had a minimal covariate missingness (1.38%–2.44%) (supplementary material Table 3). After excluding incomplete cases, 6418 children remained, of whom 9.5% (*n* = 608) were reported by mothers to have early mental health problems. Exact matching yielded a final analytic sample of 1206 observations, with a 1:1 balance between groups (*n* = 603 per group) (Fig. 1). Descriptive comparisons between matched and unmatched group showed similar background characteristics in sex, age, marital status, and number of children in the family. However, the unmatched group had higher proportions of mothers with university education (76.1% vs. 67.5%), Swedish-born mothers (86.6% vs. 80.9%), and lower parental distress (19.9% vs. 42.6%). After matching, the groups were well-balanced (Table 1), consisting of 51% boys and 49% girls, mostly recruited at 3 years of age (50.8%). Most mothers (> 95%) were either married or cohabiting. A slight imbalance remained in the number of children in the household (SMD = 0.18), with the mental health problem group had more single-child households (22.2% vs. 16.1%) while the comparison group had more households with three or more siblings (19.1% vs. 13.9%).

**Table 1:** Baseline characteristics of the study sample before and after matching (Mother-rated SDQ)

	Before matching							After matching				SMD
	Total sample (*N* = 6418)		Children with early mental health problems (*n* = 608)		Children without early mental health problems (*n* = 5810)		SMD	Children with early mental health problems (*n* = 603)		Children without early mental health problems (*n* = 603)		
	*n*°	%	*n*°	%	*n*°	%		*n*°	%	*n*°	%	
Sex												
Girls	3176	49.00%	301	49.51%	2875	49.48%	0.00046	298	49.42%	298	49.42%	0.0
Boys	3242	51.00%	307	50.49%	2935	50.52%		305	50.58%	305	50.58%	
Age												
3 years	3270	50.95%	306	50.33%	2964	51.02%	− 0.02791	306	50.75%	306	50.75%	0.0
4 years	1730	26.96%	159	26.15%	1571	27.04%		156	25.87%	156	25.87%	
5 years	1418	22.09%	143	23.52%	1275	21.94%		141	23.38%	141	23.38%	
Mean (SE)			3.73 (0.03)		3.73 (0.03)			3.73 (0.02)		3.73 (0.02)		
Level of education												
Primary school	128	1.99%	31	5.10%	97	1.67%	0.221	27	4.48%	27	4.48%	0.0
High school	1509	23.51%	170	27.96%	1339	23.05%		169	28.03%	169	28.03%	
University	4781	74.49%	407	66.94%	4374	75.28%		407	67.50%	407	67.50%	
Country of birth												
Sweden	5491	85.56%	489	80.43%	5002	86.09%	0.152	488	80.93%	488	80.93%	0.0
Other	927	14.44%	119	19.57%	808	13.91%		115	19.07%	115	19.07%	
Psychological distress												
Yes	1552	24.18%	262	43.09%	1290	22.20%	− 0.457	257	42.62%	257	42.62%	0.0
No	4866	75.82%	346	56.91%	4520	77.80%		346	57.38%	346	57.38%	
Marital status^a^												
Married/co-habiting	6174	96.20%	584	96.05%	5590	96.21%	− 0.00834	580	96.19%	573	95.02%	0.05661
Other	244	3.80%	24	3.95%	220	3.79%		23	3.81%	30	4.98%	
Number of children in the family^a^												
1	1164	18.14%	137	22.53%	1027	17.68%	0.198	134	22.22%	97	16.09%	0.18974
2	3936	61.33%	386	63.49%	3550	61.10%		385	63.85%	391	64.84%	
3+	1318	20.54%	85	13.98%	1233	21.22%		84	13.93%	115	19.07%	

*SMD* Standardized mean difference, *SE* Standard error

^a^ Adjusted in the regression models

As shown in Table 2, on average, children with early mental health problems had higher total cumulative healthcare utilization (mean: 28.96, 95%CI 25.92–32) compared to children without early mental health problems (mean: 20.17, 95%CI 17.98–22.35) within the four-year follow up. Total cumulative healthcare costs followed a similar pattern, with the early mental health problems group averaging US$4,255 (95%CI $3,701–$4,808) versus US$2,794 (95%CI $2,372–$3,215) for their counterparts.

**Table 2:** Average cumulative healthcare utilization and associated costs over 4-year-follow-up among children with and without early mental health problems (Mother-rated SDQ)

	Children with early mental health problems (*n* = 603)	Children without early mental health problems (*n* = 603)	Between-group difference
Prescribed medication			
Utilization			
n	493	463	
Mean (SE)	11.09 (0.80)	8.17 (0.67)	2.92 (1.04)
Median (IQR)	3 (1–11)	3 (1–9)	
95%CI	9.50–12.68	6.86–9.48	0.88–4.96
Costs (in USD)^a^			
Mean (SE)	624 (131)	331 (76)	293 (152)
Median (IQR)	67 (18–282)	52 (12–192)	
95%CI	366–882	182–480	− 4–590
Inpatient care			
Utilization			
n	41	26	
Mean (SE)	0.09 (0.02)	0.07 (0.02)	0.02 (0.028)
Median (IQR)	0 (0–0)	0 (0–0)	
95%CI	0.06–0.13	0.04–0.10	− 0.035–0.075
Costs (in USD)^a^			
Mean (SE)	506 (91)	347 (86)	159 (125)
Median (IQR)	0 (0–0)	0 (0–0)	
95%CI	328–685	177–517	− 86–404
Specialized outpatient care			
Utilization			
n	474	405	
Mean (SE)	3.74 (0.20)	2.48 (0.15)	1.26 (0.25)
Median (IQR)	2 (1–5)	1 (0–3)	
95%CI	3.35–4.12	2.19–2.78	0.77–1.75
Costs (in USD)^a^			
Mean (SE)	1739 (103)	1111 (73)	628 (127)
Median (IQR)	888 (357–2133)	477 (0–1371)	
95%CI	1538–1941	967–1255	380–876
Primary care			
Utilization			
n	488	484	
Mean (SE)	14.04 (1.05)	9.45 (0.60)	4.59 (1.21)
Median (IQR)	8 (2–16)	6 (1–13)	
95%CI	11.98–16.10	8.27–10.62	2.23–6.94
Costs (in USD)^a^			
Mean (SE)	1385 (90)	1004 (60)	381 (108)
Median (IQR)	777 (179–1704)	673 (135–1378)	
95%CI	1209–1560	887–1122	170–591
Total healthcare			
Utilization			
n	592	584	
Mean (SE)	28.96 (1.55)	20.17 (1.11)	8.79 (1.92)
Median (IQR)	16 (8–34)	13 (6–25)	
95%CI	25.92–32.00	17.98–22.35	5.03–12.55
Costs (in USD)^a^			
Mean (SE)	4255 (282)	2794 (215)	1461 (352)
Median (IQR)	2127 (945–4588)	1525 (715–3082)	
95%CI	3701–4808	2372–3215	771–2151

*CI* Confidence interval, *IQR* Interquartile range, *SE* Standard error

^a^ Costs in 2024 USD

The trend was consistent across all healthcare categories except for inpatient care. Children with early mental health problems had 11 units of prescribed medication (95%CI 9.5–12.68) and costed US$624 (95% CI $366–$882). They also had 4 specialized outpatient visits (95%CI 3.35–4.12), with average costs of US$1,739 (95%CI $1,538–$1,941), and 14 primary care visits (95%CI 11.98–16.10) with average costs of US$1,385 (95%CI $1,209–$1,560). However, negligibly low values were seen in both groups in terms of inpatient care visits (mean: 0.09, 95%CI 0.06–0.13 versus mean: 0.07, 95%CI 0.04–0.10), and costs (mean: $506, 95%CI $328–$685 versus mean: $347, 95%CI $177–$517).

### Incremental healthcare utilization and costs

**Fig. 2:**
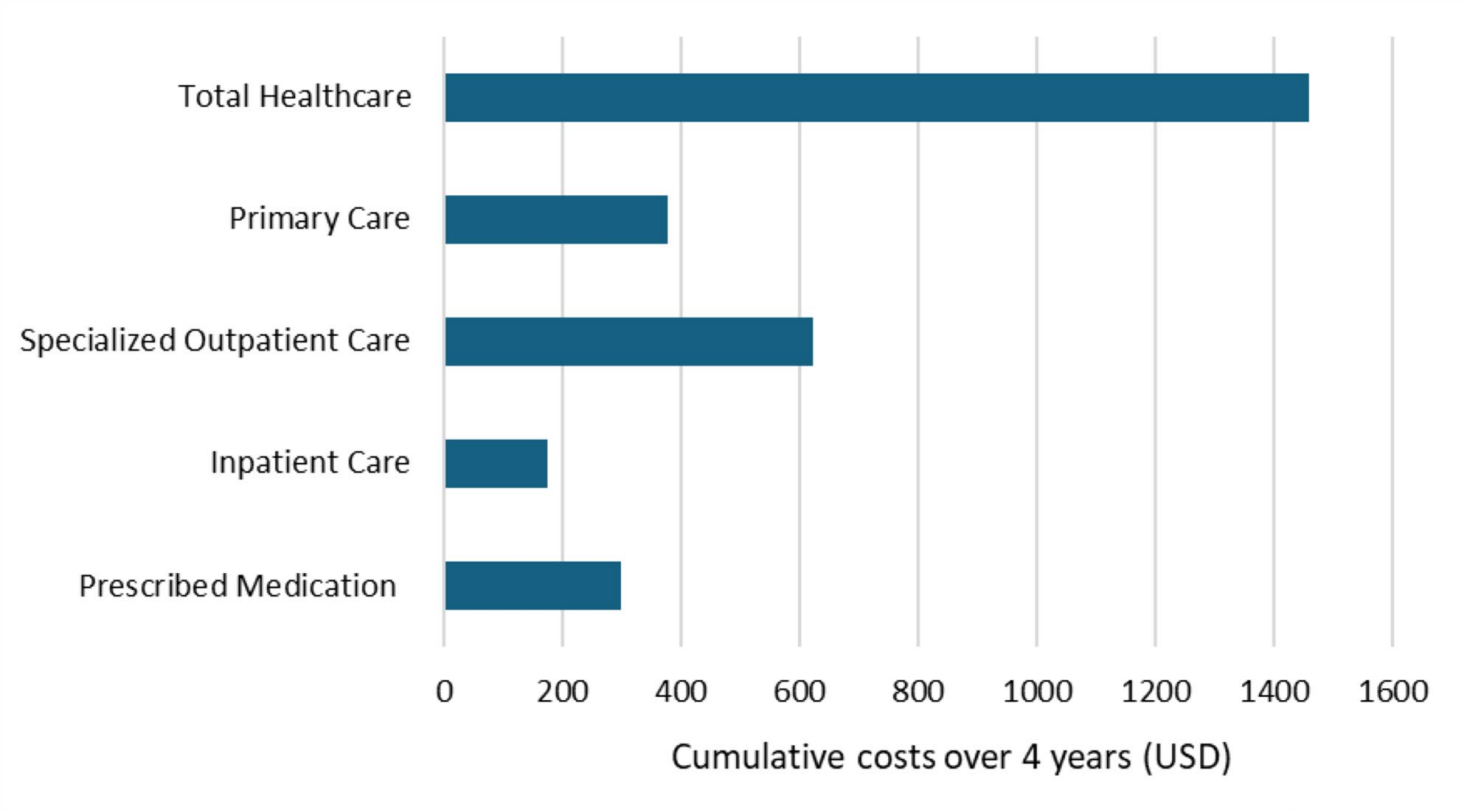
Incremental cumulative costs for children with early mental health problems over 4 years (Mother-rated SDQ)

Regression results (Table 3) showed statistically significant incremental utilization and related costs for all healthcare categories except inpatient care among children with early mental health problems compared to their counterparts. On average, a child with early mental health problems consumed approximately nine additional healthcare visits (8.84, 95%CI 5.97–11.72) and incurred US$1,457 (95%CI $741–$2,173) additional total costs over the four-year period. Moreover, they had three additional medication prescriptions (2.98, 95%CI 0.91–5.04) with associated costs of approximately US$300 ($299, 95%CI $68–$591). Similarly, primary care use and associated costs were higher among the early mental health problems group, with extra 4.6 visits (95%CI 2.23–6.9) and US$376 (95%CI $168–$585) additional costs per child. Specialized outpatient care also showed significantly higher utilization and costs among children with mental health problems, with 1.23 more visits (95%CI 0.75–1.72) and US$622 (95%CI $373–$870) in costs. Cumulative costs of different health service categories over the 4-year follow-up are displayed in Fig. 2.

**Table 3:** Incremental healthcare utilization and costs of children with early mental health problems (Mother-rated SDQ)

Children with early mental health problems (*n* = 603)	Marginal effects (95%CI)
Prescribed medication	
Utilization	2.98 (0.91, 5.04)*
Costs (in USD)^a^	299 (68–591)*
Inpatient care	
Utilization	0.03 (− 0.02, 0.07)
Costs (in USD)^a^	175 (− 69–419)
Specialized outpatient care	
Utilization	1.23 (0.75, 1.72)***
Costs (in USD)^a^	622 (373–870)***
Primary care	
Utilization	4.6 (2.23, 6.9)***
Costs (in USD)^a^	376 (168–585)***
Total healthcare	
Utilization	8.84 (5.97, 11.72)***
Costs (in USD)^a^	1457 (741–2173)***

*CI* Confidence interval

***: Statistically significant at p-value 0.001

*: Statistically significant at p-value 0.05

^a^ Costs in 2024 USD

### Psychiatric diagnoses

Cumulative healthcare utilization and costs were primarily driven by non-psychiatric related care in both groups (Table 4). Nonetheless, children with early mental health problems utilized significantly more services specifically related to psychiatric diagnoses, with a between-group mean difference of 1.17 units (95% CI 0.58–1.76) and an associated cost difference of US$184 (95% CI $56–$312) in total healthcare. In specialized outpatient and primary care settings, psychiatric-related utilization was low and mean difference was negligible, with 0.22 (95%CI 0.12–0.32) specialized outpatient visits and 0.04 (95%CI 0.02–0.06) primary care visits with associated costs of US$95 (95%CI $47–$143) and US$5 (95%CI $2–$8) respectively for children with early mental health problems compared to those without. No significant psychiatric related utilization and costs were seen in inpatient care in either group.

**Table 4:** Psychiatric and non-psychiatric related healthcare consumptions and costs (Mother-rated SDQ)

Category	Children with early mental health problems (*n* = 603)			Children without early mental health problems (*n* = 603)			Between-group difference			
	Mean (SE)	95% CI	Median (IQR)	Mean (SE)	95% CI	Median (IQR)	Mean	SE	Lower CI	Upper CI
Prescribed medication										
Utilization										
Neuroleptics (N05)	0.44 (0.11)	0.22, 0.66	0 (0–0)	0.17 (0.06)	0.05, 0.28	0 (0–0)	0.27	0.13	0.02	0.52
Psychoanaleptics (N06)	0.72 (0.17)	0.39, 1.05	0 (0–0)	0.08 (0.05)	− 0.01, 0.17	0 (0–0)	0.64	0.18	0.29	0.99
Other medications	9.93 (0.74)	8.47, 11.39	3 (1–10)	7.92 (0.66)	6.64, 9.20	3 (1–9)	2.01	0.99	0.07	3.95
Total	11.09 (0.80)	9.50, 12.68	3 (1–11)	8.17 (0.67)	6.86, 9.48	3 (1–9)	2.92	1.04	0.87	4.97
Costs (USD)^a^										
Neuroleptics (N05)	37 (10)	17, 57	0 (0–0)	6 (2)	2, 11	0 (0–0)	31	10.2	11.01	51
Psychoanaleptics (N06)	70 (24)	24, 117	0 (0–0)	8 (5)	− 2, 17	0 (0–0)	62	25	14	110
Other medications	517 (126)	269, 765	61 (15–224)	317 (76)	169, 465	52 (10–183)	200	147	− 88	488
Total	624 (131)	366, 882	67 (18–282)	331 (76)	182, 480	52 (12–192)	293	152	− 4	590
Inpatient care										
Utilization										
Psychiatric care	0.002 (0.002)	− 0.002, 0.005	0 (0–0)	0.005 (0.005)	− 0.005, 0.01	0 (0–0)	− 0.00	0.01	− 0.01	0.01
Non-psychiatric care	0.09 (0.02)	0.06, 0.12	0 (0–0)	0.06 (0.01)	0.03, 0.09	0 (0–0)	0.03	0.02	− 0.01	0.07
Total	0.09 (0.02)	0.06, 0.13	0 (0–0)	0.07 (0.02)	0.04, 0.10	0 (0–0)	0.02	0.03	− 0.03	0.08
Costs (USD)^a^										
Psychiatric care	18 (18)	− 18, 54	0 (0–0)	27 (27)	− 26, 81	0 (0–0)	− 9	33	− 73	55
Non-psychiatric care	437 (82)	277, 597	0 (0–0)	266 (65)	139, 393	0 (0–0)	171	105	− 36	378
Total	506 (91)	328, 685	0 (0–0)	347 (86)	177, 517	0 (0–0)	159	125	− 86	404
Specialized outpatient care										
Utilization										
Psychiatric care	0.30 (0.04)	0.23, 0.37	0 (0–0)	0.08 (0.03)	0.02, 0.15	0 (0–0)	0.22	0.05	0.12	0.32
Non-psychiatric care	3.27 (0.17)	2.93, 3.60	2 (1–4)	2.28 (0.13)	2.02, 2.53	1 (0–3)	0.99	0.21	0.59	1.39
Total	3.74 (0.20)	3.35, 4.12	2 (1–5)	2.48 (0.15)	2.19, 2.78	1 (0–3)	1.26	0.25	0.77	1.75
Costs (USD)^a^										
Psychiatric care	137 (17)	104, 170	0 (0–0)	42 (18)	6, 78	0 (0–0)	95	25	47	143
Non-psychiatric care	1520 (91)	1341, 1700	794 (343–1952)	1007 (61)	886, 1127	456 (0–1338)	513	109	299	727
Total	1739 (103)	1538, 1941	888 (357–2133)	1111 (73)	967, 1255	477 (0–1371)	628	127	380	876
Primary care										
Utilization										
Psychiatric care	0.05 (0.01)	0.03, 0.08	0 (0–0)	0.01 (0.01)	0.003, 0.02	0 (0–0)	0.04	0.01	0.02	0.06
Non-psychiatric care	1.17 (0.08)	1.01, 1.34	0 (0–2)	1.11 (0.08)	0.99, 1.30	0 (0–2)	0.06	0.11	− 0.15	0.27
Total	14.04 (1.05)	11.98, 16.10	8 (2–16)	9.45 (0.60)	8.27, 10.62	6 (1–13)	4.59	1.21	2.23	6.94
Costs (USD)^a^										
Psychiatric care	6 (1)	3, 9	0 (0–0)	1 (1)	0.1, 2	0 (0–0)	5	1	2	8
Non-psychiatric care	157 (11)	135, 179	0 (0–270)	152 (11)	131, 173	0 (0–224)	5	16	− 25	35
Total	1385 (90)	1209, 1560	777 (179–1704)	1004 (60)	887, 1122	673 (135–1378)	381	108	170	592
Total healthcare										
Utilization										
Psychiatric care	1.52 (0.28)	0.97, 2.06	0 (0–0)	0.35 (0.11)	0.13, 0.57	0 (0–0)	1.17	0.3	0.58	1.76
Non-psychiatric care	14.46 (0.87)	12.74, 16.17	7 (3–16)	11.39 (0.76)	9.90, 12.89	5 (2–14)	3.07	1.15	0.81	5.33
Total	28.96 (1.55)	25.92, 32.00	16 (8–34)	20.17 (1.11)	17.98, 22.35	13 (6–25)	8.79	1.92	5.03	12.55
Costs (USD)^a^										
Psychiatric care	269 (47)	177, 360	0 (0–0)	85 (46)	− 5, 175	0 (0–0)	184	65	56	312
Non-psychiatric care	2631 (240)	2159, 3102	1064 (418–2707)	1742 (153)	1441, 2042	739 (174–1911)	889	285	330	1448
Total	4255 (282)	3701, 4808	2127 (945–4588)	2794 (215)	2372, 3215	1525 (715–3082)	1461	352	771	2151

*CI* Confidence interval, *IQR* Interquartile range, *SE* Standard error

^a^ Costs in 2024 USD

For prescribed medications, both neuroleptics (N05) and psycho-analeptics (N06) were prescribed less frequently than other medication types in both groups, with between-group mean difference showing 0.27 (95%CI 0.02–0.52) for neuroleptics, and 0.64 (95%CI 0.29–0.99) for psycho-analeptics. Corresponding costs showed slightly higher mean value for children with early mental health problems, with a mean difference of US$31 (95%CI $11.01–$51) for neuroleptics and US$62 (95%CI $14–$110) for psycho-analeptics. No consumption of other nervous system drugs (N07) was detected.

The top five most commonly prescribed medications were dominated by drugs for constipation (A06), obstructive airway diseases (R03), and antibiotics for systematic use (J01) in both groups. Psycho-analeptics (N06) appeared among the top five medications only for children with early mental health problems, whereas antihistamines (R06) were more frequent in the comparison group (Supplementary material Fig. 3).

The top five psychiatric and non-psychiatric related ICD-10 diagnoses for each group are shown in Figs. 4 and 5 in supplementary material. All non-psychiatric related diagnoses were identical in both groups with health examination and investigation being the most frequent, followed by asthma. In contrast, hyperkinetic disorders (F90) and pervasive developmental disorders (F84) were most commonly diagnosed in problems group, while non-specific other behavioral and emotional disorders (F98) and conduct disorders (F91) were two most common diagnoses in the healthy peer group.

### Sub-group analyses

Among children with early mental health problems, girls and boys did not differ significantly in inpatient care or medication use. However, girls utilized significantly less primary care, specialized outpatient care and overall healthcare compared to boys with mean differences of − 5.16 (95%CI − 9.24 to − 1.09), − 0.82 (95%CI − 1.58 to − 0.06), and − 7.32 (95%CI − 13.35 to − 1.29), respectively. They also incurred significantly lower primary care costs, with a between-group mean difference of - US$416 (95%CI − $764 to − $68). Differences in costs were not significant in inpatient care and medication.

While stratified by symptom profiles, boys with externalizing problems had significantly higher utilization and costs compared to girls across all health service categories with mean values of 13.41 units of prescribed medication (95%CI 9.25–17.56), 3.66 specialized outpatient care visits (95%CI 2.83–4.50), 11.91 primary care visits (95%CI 8.95–14.87), and 29.07 total healthcare visits (95%CI 22.74–35.40). Mean costs were US$823 for medications (95%CI $354–$1,291), US$1,642 for specialized outpatient (95%CI $1,242–$2,042), US$1,240 for primary care (95%CI $949–$1,531), and US$4,089 for total (95%CI $3,002–$5,175). A similar trend was observed among boys with internalizing problems. Compared to their female counterparts, they had higher mean values ranging from 12.68 units of prescribed medication (95%CI 7.71–17.65) and US$565 (95% CI $220–$909) related costs, 4.05 outpatient care visits (95% CI 2.97–5.14) and US$1,935 (95% CI $1,322–$2,548) related costs, 21.04 primary care visits (95% CI 9.89–32.19) and US$1,821 (95% CI $978–$2,663) related costs, and 37.84 total healthcare visits (95% CI 23.90–51.77) and US$4,740 (95% CI $3,114–$6,366) related costs.

Detailed results on sub-group analyses can be found in the supplementary material Tables 4 and 5.

### Sensitivity analyses

Total sample with complete information were 6257 for father-raters and 5288 for teacher-raters before matching. Covariate missingness was small (1%–3%) for the fathers’ group and there was no missingness in the teachers’ group. Both raters had a higher proportion of children with early mental health problems compared to mothers (9.5%), with 12.8% for fathers and 10.1% for teachers. The father-rating group had a slightly higher proportion of girls (50.56%) than boys (49.44%). In the teachers’ group, boys were 3% higher than girls (51.40% vs 48.60%). The median age was 3 years old with IQR 3–4 in both groups. As in the base case, more than half (56%) of the father respondents had a university education, a total of 84% were born in Sweden, and more than 95% were either married or in partnership. Total analytic samples after matching were reduced to 801 in each group in fathers-rating sample and 535 in teachers’ respectively with 1:1 balance between the treatment and comparison groups.

Regression analyses using father-rated SDQ showed similar results to that of mothers’ rating. According to fathers’ ratings, children with early mental health problems had significantly higher incremental healthcare utilization and costs in terms of prescribed medication (3.16, 95% CI 1.49–4.83; US$331, 95% CI $77–$584), specialized outpatient care (1.06, 95% CI 0.59–1.53; US$563, 95% CI $313–$813), primary care (3.57, 95% CI 1.91–5.23; US$323, 95% CI $172–$473), and total healthcare (7.91, 95% CI 5.65–10.18; US$1,563, 95% CI $912–$2,213). All results were statistically significant.

Teachers’ ratings also indicated significantly larger consumption and costs related to specialized outpatient care (0.89, 95% CI 0.23–1.54; US$448, 95% CI $124–$771), primary care (3.26, 95% CI 1.09–5.42; US$272, 95% CI $71–$473), and total healthcare (6.32, 95% CI 3.17–9.48; US$1,255, 95% CI $524–$1,986). However, incremental use of prescribed medication and inpatient care were not statistically significant.

Detailed results can be found in the supplementary material Tables 6 and 7.

## Discussion

This longitudinal observational study used a large cohort of over 6000 children aged 3–5 years old from the Uppsala Region in Sweden. To our knowledge, this is the first study to explore the healthcare utilization and associated costs of early mental health problems among preschool children in Sweden. This study showed that children with early mental health problems consumed significantly larger healthcare resources and incurred higher costs across multiple healthcare services over a four-year period, compared to those without early mental health problems.

Findings based on mother-rated SDQ indicated that children with early mental health problems had nine additional healthcare visits and costs of US$1,457. The largest consumption of healthcare resources was seen in primary care with almost five additional visits followed by three additional units of prescribed medication, and one additional specialized outpatient visit. This could be due to the fact that primary care is the first line of health service contact in Sweden, and that referral from primary care is usually needed for timely access to the specialist healthcare [19]. A large part of healthcare expenditure (US$622) was seen in specialized outpatient care. In Sweden, specialized outpatient care includes both specialized somatic and psychiatric healthcare visits to physicians, nurses, psychologists, social-workers, occupational therapists, and physiotherapists, which are resource intensive. Moreover, since the healthcare reform in Sweden in the 1990s, inpatient care has been increasingly outsourced to specialized outpatient care [19, 44]. This also explains the mass of null consumptions and costs in inpatient care in relation to outpatient care.

The results were similar to previous research from other countries. An annual report on healthcare utilization and expenditures for children with mental health conditions using a nationwide sample in the US showed that children aged 1–17 years old with mental health problems had significant increase in healthcare visits by 50%, with a total of US$11.6 billion expenditure between 2006 and 2011 [11]. Likewise, a national survey on economic impact of childhood mental health illness from the UK showed that children aged 5–15 years old with diagnosed psychiatric disorders had substantial annual utilization costs, ranging from 15.8 million pounds in primary care to 64.2 million pounds in specific mental health services [7].

Setting our findings in the larger framework of the long-term economic impact of child mental health issues is also crucial. According to Wellander et al. (2016), school-aged children in Sweden who had psychosocial, anxiety, or depression needed extra help in school, costing between US$ 8,855 and US$ 37,229 a month [45]. This shows how quickly the expenses of the education sector alone can surpass the early healthcare costs estimated in this study. Although Swedish data remain scarce, international studies provide further perspective. For example, a US study estimated mean annual total costs of US$ 6,405 per child with anxiety or depression in 2016, including both direct and indirect costs [46]. Taken together, these findings suggest that the additional healthcare costs we identified in preschool represent only a fraction of the potential long-term impact.

In our study, non-psychiatric related care was the largest driver of utilization and related costs in both children with and without early mental health problems. This could be explained by the follow-up period. Children were only followed for 4 years from the age of 3–5 years, which could be too short to capture the possible diagnoses of psychiatric illness, which are usually more frequent in the later childhood. According to the WHO, the prevalence of certain mental health problems, such as depression, anxiety, and eating disorders increase with increasing age, with most prevalence in the 10–19 years old group [47]. In addition, findings from previous research [12, 13, 48, 49] showed that higher consumption and costs related to mental health problems were more pronounced in young adolescents aged 12–17.

Stratified analysis by sex showed that boys with early mental health problems consumed more primary care and specialized outpatient care compared to girls. In previous research, which used data on children and young adolescents aged 3–17 years from Region Västra Götaland, Sweden, higher primary care visits were seen among girls compared to boys. The contrasting results could be explained by the age group of study population. The previous study included adolescent population where girls are a more prevalent group with depression and anxiety, and primary care in Sweden is usually the first line of service to go [21, 50].

Boys with externalizing symptoms had more healthcare visits and incurred higher costs than girls. This finding is similar to previous studies where hyperactive male preschoolers were the only predictor of additional costs and utilization [8]. Ample literature showed that externalizing problems are more commonly associated with boys (e.g., ADHD, conduct disorders) and that boys are more likely to externalize their symptoms such as showing more impulsive and aggressive behavior [51–55]. In addition, our study found that boys with internalizing problems utilized more healthcare services than their peers, with slightly higher use than those with externalizing problems, particularly in primary care and specialized outpatient care. Traditionally, boys are more commonly associated with externalizing problems and girls with internalizing problems. However, our findings reflect a previous study in Sweden looking at the trends on internalizing symptoms of childhood and adolescent, where there were significantly increasing percentage of boys with anxiety symptoms and self-reported internalizing symptoms [56].

Among the three different raters, fathers had higher ratings of their children’s SDQ total difficulties scores compared to mothers (13% vs. 9%). This could be due to several factors, such as differing interactions between fathers and mothers with their children, or mothers being more often the primary caregivers which may lead to them becoming desensitized to their children’s problem behaviors [24]. Despite that, our multi-rater analyses yielded similar findings between both parents.

Our study has strengths and limitations. A key strength is that the study used multi-rater assessments which reduces single-informant bias, captures context-specific symptoms across home and school settings, and thus enhances the validity and policy relevance of findings within the Swedish child healthcare and education settings. Moreover, this is the first study in Sweden to explore the longer-term economic impact of preschool children with early mental health problems. In addition, the study used observational longitudinal data with linkage to the National Patient Registers and regional data, which is a key strength as it allows for comprehensive and accurate tracking of healthcare utilization over time across different care settings. This linkage enables the inclusion of real-world, routinely collected data at both national and regional levels, reducing recall bias, enhancing generalizability, and supporting a more complete estimation of healthcare costs and outcomes. Additionally, the sample size is relatively large compared to previous research [20]. Regarding the limitations, the study might underestimate the healthcare utilization and associated costs of early childhood mental health problems due to relatively short follow-up time. Some negative consequences, such as the development of more severe psychiatric disorders and increased healthcare use, may not fully emerge until adolescence or adulthood [57]. A longer follow-up would likely capture a greater portion of the long-term individual, societal, and economic impacts. Moreover, our study lacked a few socio-economic variables such as household income and residential area that could have been included in the matching. While our study captured the earliest SDQ assessment, the majority of the cohort had only one assessment. Consequently, our analysis primarily reflected early mental health problems assessed on one occasion. This data structure precluded the stratification of the data sample into ‘persistent’ versus ‘transient’ groups, which would require repeated SDQ measurements over time to verify the stability of the mental health problems in children. Lastly, although generalizability is a possibility within a similar region context, cautions should be taken when applying the results to another country context outside of Sweden.

Our study has substantial public health implications. On scaling our results to a national level, if 6.7% of the country’s preschool population (about 350,000 children aged 3 to 5) screen positive for early mental health issues, this would correspond to roughly 23,499 impacted children in Sweden in 2024. Then the 4-year incremental healthcare expenditure ranges from 4.11 million USD (36.33 million SEK) in inpatient care to 34.24 million USD (302.48 million SEK) of total healthcare expenditure at the population level, with specialized outpatient care contributing to more than one third of the total healthcare costs (14.62 million USD/129.13 million SEK) (see detailed calculation in Table 8 supplementary material). In comparison, Sweden’s national healthcare budget in 2024 was 13.13 billion USD (116 billion SEK), which means that our 4-year cost estimates would correspond to approximately 0.03% to 0.26% of the annual national healthcare spending attributable to preschool children with early mental health problems. While this represents only about 0.2% of Sweden’s national healthcare budget for 2024, it is striking given that these costs arise solely from preschool children and only reflect healthcare expenditures. Considering the likelihood of increased costs during school years and the additional impact on education and social services, the long-term economic impact is likely far greater. Healthcare planners and policy makers of Sweden, particularly Uppsala Region, should be informed of the economic impact of early mental health problems and the importance of investing in interventions for early diagnosis, and effective, and timely care among preschool children.

While our study did not measure help-seeking behaviors or treatment gaps, it is important to contextualize these findings within the Swedish healthcare sector landscape. In Sweden, Child Health Centers (CHC), where 95% of 3–5 year-olds are enrolled, serve as the primary entry points for early detection [58, 59]. Thus, the CHC represent critical settings where early interventions should be targeted. While national guidelines emphasize the importance of early interventions targeting mental health problems [60], Sweden currently lacks a systematic, nationwide screening protocol for procedure for mental health problems within the CHC. However, regional implementations of the SDQ for the screening of mental health problems withing the CHC (such as in Uppsala and Stockholm) [61] and the nationwide rollout of evidence-based parenting programs [62] represent significant policy efforts to bridge these treatment gaps and provide support before symptoms escalate.

Future research should be conducted with a longer follow-up period to capture the adolescents’ mental health problems impact, healthcare utilization and associated costs. Moreover, our estimates reflect direct healthcare costs only. We did not include indirect costs such as productivity losses of parents/caregivers to care for their children, or other sector costs such as education/social service costs. Inclusion of these costs would likely increase the estimated economic impact substantially. It would also be relevant to conduct a similar study including on a national level in Sweden and compare with our findings. Such comparisons could help identify inequities, inform regional planning, and support more targeted policy interventions.

## Conclusions

This study highlights the significant impact associated with early childhood mental health problems over a four-year period in Sweden, particularly in primary care, specialized outpatient care, and prescribed medications. The presence of additional healthcare utilization from as early as age three underscores the preschool period as critical window for early identification and targeted intervention with potential for cost-offsetting within the healthcare system. The consistency of findings across parents- and teacher-reports further supports the robustness of multi-informant assessment as a policy-relevant approach for identifying children at risk of high healthcare utilization. Future research with longer follow-up and comparison with national Swedish estimates is needed to explore variation in care patterns and costs, and to inform effective mental health service planning across the country. Additionally, cross-national comparisons would offer insightful information about how Sweden’s healthcare utilization patterns and related expenses of early childhood mental health problems align with or deviate from international contexts.

## Supplementary Information

Below is the link to the electronic supplementary material.

Supplementary Material 1.

## Data Availability

The corresponding author can be contacted for any data not included in the article or supplementary material. Data will be provided based on the nature of the research request.

## References

[1] Polanczyk GV, Salum GA, Sugaya LS, Caye A, Rohde LA. Annual research review: a meta-analysis of the worldwide prevalence of mental disorders in children and adolescents. J Child Psychol Psychiatry. 2015;56:345–65. 10.1111/JCPP.12381. 25649325 10.1111/jcpp.12381

[2] Vasileva M, Graf RK, Reinelt T, Petermann U, Petermann F. Research review: a meta-analysis of the international prevalence and comorbidity of mental disorders in children between 1 and 7 years. J Child Psychol Psychiatry. 2021;62:372–81. 10.1111/JCPP.13261. 32433792 10.1111/jcpp.13261

[3] Currie J, Stabile M, Manivong P, Roos LL. Child health and young adult outcomes. J Hum Resour. 2010;45:517–48. 10.1353/JHR.2010.0013.

[4] Fergusson DM, Horwood LJ, Ridder EM. Show me the child at seven: the consequences of conduct problems in childhood for psychosocial functioning in adulthood. J Child Psychol Psychiatry Allied Discip. 2005;46:837–49. 10.1111/J.1469-7610.2004.00387.X. 10.1111/j.1469-7610.2004.00387.x 16033632

[5] Patel V, Flisher AJ, Hetrick S, McGorry P. Mental health of young people: a global public-health challenge. Lancet. 2007;369:1302–13. 10.1016/S0140-6736(07)60368-7. 17434406 10.1016/S0140-6736(07)60368-7

[6] Lawrence D, Dawson V, Houghton S, Goodsell B, Sawyer MG. Impact of mental disorders on attendance at school. Aust J Educ. 2019;63:5–21. 10.1177/0004944118823576/ASSET/025C45BD-6DDC-4391-9B9A-6EAEFA90EEFE/ASSETS/IMAGES/LARGE/10.1177_0004944118823576-FIG1.JPG.

[7] Snell T, Knapp M, Healey A, Guglani S, Evans-Lacko S, Fernandez JL, Meltzer H, Ford T. Economic impact of childhood psychiatric disorder on public sector services in Britain: estimates from national survey data. J Child Psychol Psychiatry. 2013;54:977–85. 10.1111/JCPP.12055. 23442096 10.1111/jcpp.12055

[8] Chorozoglou M, Smith E, Koerting J, Thompson MJ, Sayal K, Sonuga-Barke EJS. Preschool hyperactivity is associated with long-term economic burden: evidence from a longitudinal health economic analysis of costs incurred across childhood, adolescence and young adulthood. J Child Psychol Psychiatry. 2015;56:966. 10.1111/JCPP.12437. 26072954 10.1111/jcpp.12437 PMC4744758

[9] Gandhi S, Chiu M, Lam K, Cairney JC, Guttmann A, Kurdyak P. Mental health service use among children and youth in Ontario: population-based trends over time. Can J Psychiatry. 2016;61:119–24. 10.1177/0706743715621254/SUPPL_FILE/SUPPL-APPENDIX.PDF. 27253703 10.1177/0706743715621254 PMC4784237

[10] Ziebold C, Silva-Ribeiro W, King D, McDaid D, Hoffmann MS, Romeo R, Pan PM, Miguel EC, Bressan RA, Rohde LA, Salum GA, de Mari J, Evans-Lacko J. Utilisation and costs of mental health-related service use among adolescents. PLoS ONE. 2022. 10.1371/JOURNAL.PONE.0273628. e0273628. 36084089 10.1371/journal.pone.0273628 PMC9462733

[11] Torio CM, Encinosa W, Berdahl T, McCormick MC, Simpson LA. Annual report on health care for children and youth in the United States: national estimates of cost, utilization and expenditures for children with mental health conditions. Acad Pediatr. 2015;15:19–35. 10.1016/J.ACAP.2014.07.007. 25444653 10.1016/j.acap.2014.07.007

[12] Saunders NR, Gandhi S, Chen S, Vigod S, Fung K, De Souza C, Saab H, Kurdyak P. Health care use and costs of children, adolescents, and young adults with somatic symptom and related disorders. JAMA Netw Open. 2020. 10.1001/jamanetworkopen.2020.11295. e2011295. 32701161 10.1001/jamanetworkopen.2020.11295 PMC7378752

[13] Larrañaga I, Ibarrondo O, Mar-Barrutia L, Soto-Gordoa M, Mar J. Excess healthcare costs of mental disorders in children, adolescents and young adults in the Basque population registry adjusted for socioeconomic status and sex. Cost Eff Resour Alloc. 2023;21:1–12. 10.1186/S12962-023-00428-W/TABLES/6. 36859271 10.1186/s12962-023-00428-w PMC9975849

[14] Smith JP, Smith GC. Long-term economic costs of psychological problems during childhood. Soc Sci Med. 2010;71:110–5. 10.1016/j.socscimed.2010.02.046. 20427110 10.1016/j.socscimed.2010.02.046 PMC2887689

[15] Lee NBC, Fung DSS, Teo J, Chan YH, Cai YM. Five-year review of adolescent mental health usage in Singapore. Ann Acad Med Singap. 2003;32:7–11. 10.47102/ANNALS-ACADMEDSG.V32N1P7. 12625091

[16] Beecham J. Annual research review: child and adolescent mental health interventions: a review of progress in economic studies across different disorders. J Child Psychol Psychiatry. 2014;55:714–32. 10.1111/JCPP.12216. 24580503 10.1111/jcpp.12216 PMC4657502

[17] Självrapporterad stress. somatiska och psykiska besvär bland skolbarn—Folkhälsomyndigheten. https://www.folkhalsomyndigheten.se/publikationer-och-material/publikationsarkiv/s/sjalvrapporterad-stress-somatiska-och-psykiska-besvar-bland-skolbarn/

[18] Skolbarns hälsovanor i Sverige. 2013/2014—Grundrapport—Folkhälsomyndigheten, https://www.folkhalsomyndigheten.se/publikationer-och-material/publikationsarkiv/s/skolbarns-halsovanor-i-sverige-201314/

[19] Sweden. health system review 2023. https://eurohealthobservatory.who.int/publications/i/sweden-health-system-review-2023

[20] Sampaio F, Ssegonja R, Nystrand C, Feldman I. Health, public sector service use and related costs of Swedish preschool children: results from the Children and Parents in Focus trial. Eur Child Adolesc Psychiatry. 2019;28:43–56. 10.1007/S00787-018-1185-1/TABLES/5. 29926252 10.1007/s00787-018-1185-1 PMC6349965

[21] Agnafors S, Kjellström AN, Björk MP, Rusner M, Torgerson J. Health care utilization in children and adolescents with psychiatric disorders. Acta Psychiatr Scand. 2023;148:327–37. .13590;JOURNAL:JOURNAL:16000447;WGROUP:STRING:PUBLICATION. 37415523 10.1111/acps.13590

[22] Kessler RC, Berglund P, Demler O, Jin R, Merikangas KR, Walters EE. Lifetime prevalence and age-of-onset distributions of DSM-IV disorders in the national comorbidity survey replication. Arch Gen Psychiatry. 2005;62:593–602. 10.1001/ARCHPSYC.62.6.593. 15939837 10.1001/archpsyc.62.6.593

[23] Barns och ungdomars. psykiska hälsa i Sverige: en systematisk litteraturöversikt med tonvikt på förändringar över tid. https://www.researchgate.net/publication/233382557_Barns_och_ungdomars_psykiska_halsa_i_Sverige_en_systematisk_litteraturoversikt_med_tonvikt_pa_forandringar_over_tid

[24] Davé S, Nazareth I, Senior R, Sherr L. A comparison of father and mother report of child behaviour on the strengths and difficulties questionnaire. Child Psychiatry Hum Dev. 2008;39:399–413. 10.1007/S10578-008-0097-6/METRICS. 18266104 10.1007/s10578-008-0097-6

[25] Salari R, Fabian H, Prinz R, Lucas S, Feldman I, Fairchild A, Sarkadi A. The children and parents in focus project: a population-based cluster-randomised controlled trial to prevent behavioural and emotional problems in children. BMC Public Health. 2013;13:1–8. 10.1186/1471-2458-13-961/TABLES/1. 24131587 10.1186/1471-2458-13-961 PMC4016486

[26] Goodman R. The strengths and difficulties questionnaire: a research note. J Child Psychol Psychiatry Allied Discip. 1997;38:581–6. 10.1111/j.1469-7610.1997.tb01545.x. 10.1111/j.1469-7610.1997.tb01545.x 9255702

[27] Pevalin DJ. Multiple applications of the GHQ-12 in a general population sample: an investigation of long-term retest effects. Soc Psychiatry Psychiatr Epidemiol. 2000;35:508–12. 10.1007/s001270050272. 11197926 10.1007/s001270050272

[28] Lundin A, Hallgren M, Theobald H, Hellgren C, Torgén M. Validity of the 12-item version of the General Health Questionnaire in detecting depression in the general population. Public Health. 2016;136:66–74. 10.1016/J.PUHE.2016.03.005. 27040911 10.1016/j.puhe.2016.03.005

[29] Dahlberg A, Fält E, Ghaderi A, Sarkadi A, Salari R. Swedish norms for the Strengths and Difficulties Questionnaire for children 3–5 years rated by parents and preschool teachers. Scand J Psychol. 2020;61:253–61. 10.1111/SJOP.12606. 31833080 10.1111/sjop.12606 PMC7079007

[30] Fält E, Fabian H, Durbeej N. Parental sociodemographic characteristics and mental health referrals by nurses in Swedish child health centres. Acta Paediatr. 2022;111:1743–51. 10.1111/APA.16448. 35673845 10.1111/apa.16448 PMC9545827

[31] Ashok P, Fäldt A, Dahlberg A, Durbeej N. Early emotional and behavioural problems predict use of habilitation services among children: Findings from a longitudinal follow-up study. PLoS ONE. 2024;19:e0303685. 10.1371/JOURNAL.PONE.0303685. 38753629 10.1371/journal.pone.0303685 PMC11098387

[32] Patient register—National Board of Health and, Welfare. https://www.socialstyrelsen.se/statistik-och-data/register/patientregistret/

[33] Prospektiva viktlistor och statistik om NordDRG -, Socialstyrelsen. https://www.socialstyrelsen.se/statistik-och-data/klassifikationer-och-koder/drg/viktlistor/

[34] KPP Databas | SKR. https://skr.se/skr/halsasjukvard/ekonomiavgifter/kostnadperpatientkpp/kppdatabas.46722.html

[35] Konsumentprisindex. (1980 = 100), skuggindextal samt index för huvudgrupper, årsmedelvärden. https://www.scb.se/hitta-statistik/statistik-efter-amne/priser-och-ekonomiska-tendenser/priser/konsumentprisindex-kpi/pong/tabell-och-diagram/konsumentprisindex-kpi/kpi-huvudgrupper-ar/

[36] CCEMG - EPPI-Centre. Cost Converter v.1.4. https://eppi.ioe.ac.uk/costconversion/

[37] Andersen RM. Revisiting the behavioral model and access to medical care: does it matter? J Health Soc Behav. 1995;36:1–10. 10.2307/2137284. 7738325

[38] RPubs. - Exact matching using R - MatchIt package. https://rpubs.com/mbounthavong/Exact_matching_MatchIt_package

[39] Deb P, Norton EC. Modeling health care expenditures and use. Annu Rev Public Health. 2018;39:489–505. 10.1146/ANNUREV-PUBLHEALTH-040617-013517/CITE/REFWORKS. 29328879 10.1146/annurev-publhealth-040617-013517

[40] Breusch TS, Pagan AR. A simple test for heteroscedasticity and random coefficient variation. Econometrica. 1979;47:1287. 10.2307/1911963.

[41] Manning WG, Mullahy J. Estimating log models: to transform or not to transform? J Health Econ. 2001;20:461–94. 10.1016/S0167-6296(01)00086-8. 11469231 10.1016/s0167-6296(01)00086-8

[42] Buntin MB, Zaslavsky AM. Too much ado about two-part models and transformation? Comparing methods of modeling Medicare expenditures. J Health Econ. 2004;23:525–42. 10.1016/J.JHEALECO.2003.10.005. 15120469 10.1016/j.jhealeco.2003.10.005

[43] Barber J, Thompson S. Multiple regression of cost data: use of generalised linear models. J Heal Serv Res Policy. 2004;9:197–204. 10.1258/1355819042250249. 10.1258/1355819042250249 15509405

[44] Tiainen A, Edman G, Flyckt L, Tomson G. Regional variations and determinants of direct psychiatric costs in Sweden. Scand J Public Health. 2008;36:483–92. 10.1177/1403494808089065. 18635731 10.1177/1403494808089065

[45] Wellander L, Wells MB, Feldman I. Does prevention pay? Costs and potential cost-savings of school interventions targeting children with mental health problems—PubMed. https://pubmed.ncbi.nlm.nih.gov/27453456/27453456

[46] Pella JE, Slade EP, Pikulski PJ, Ginsburg GS. Pediatric anxiety disorders: a cost of illness analysis. J Abnorm Child Psychol. 2020;48:551–9. 10.1007/S10802-020-00626-7/TABLES/5. 32078089 10.1007/s10802-020-00626-7

[47] Mental health of adolescents. https://www.who.int/news-room/fact-sheets/detail/adolescent-mental-health

[48] Dickson KS, Stadnick NA, Lind T, Trask EV. Defining and predicting high cost utilization in children’s outpatient mental health services. Adm Policy Ment Health. 2020;47:655. 10.1007/S10488-019-00988-1. 31701293 10.1007/s10488-019-00988-1 PMC7202946

[49] Knapp M, Snell T, Healey A, Guglani S, Evans-Lacko S, Fernandez JL, Meltzer H, Ford T. How do child and adolescent mental health problems influence public sector costs? Interindividual variations in a nationally representative British sample. J Child Psychol Psychiatry Allied Discip. 2015;56:667–76. 10.1111/JCPP.12327;REQUESTEDJOURNAL:JOURNAL:14697610;WGROUP:STRING:PUBLICATION. 10.1111/jcpp.12327 25265159

[50] Mental health among youth. in Sweden, editor, https://www.norden.org/en/publication/mental-health-among-youth-sweden

[51] Storvoll EE, Wichstrøm L. Do the risk factors associated with conduct problems in adolescents vary according to gender? J Adolesc. 2002;25:183–202. 10.1006/JADO.2002.0460. 12069434 10.1006/jado.2002.0460

[52] Hoffmann ML, Powlishta KK, White KJ. An examination of gender differences in adolescent adjustment: the effect of competence on gender role differences in symptoms of psychopathology. Sex Roles. 2004;50:795–810. 10.1023/B:SERS.0000029098.38706.B1/METRICS.

[53] Chi X, Cui X. Externalizing problem behaviors among adolescents in a southern city of China: gender differences in prevalence and correlates. Child Youth Serv Rev. 2020;119:105632. 10.1016/J.CHILDYOUTH.2020.105632.

[54] Lau TWI, Lim CG, Acharryya S, Lim-Ashworth N, Tan YR, Fung SSD. Gender differences in externalizing and internalizing problems in Singaporean children and adolescents with attention-deficit/hyperactivity disorder. Child Adolesc Psychiatry Ment Health. 2021;15:1–11. 10.1186/S13034-021-00356-8/TABLES/4. 33482840 10.1186/s13034-021-00356-8 PMC7825195

[55] White R, Renk K. Externalizing behavior problems during adolescence: an ecological perspective. J Child Fam Stud. 2012;21:158–71. 10.1007/S10826-011-9459-Y/TABLES/4.

[56] Durbeej N, Sörman K, Norén Selinus E, Lundström S, Lichtenstein P, Hellner C, Halldner L. Trends in childhood and adolescent internalizing symptoms: results from Swedish population based twin cohorts. BMC Psychol. 2019;7:1–10. 10.1186/S40359-019-0326-8/FIGURES/2. 31375136 10.1186/s40359-019-0326-8 PMC6679471

[57] Solmi M, Radua J, Olivola M, Croce E, Soardo L, Salazar de Pablo G, Il Shin J, Kirkbride JB, Jones P, Kim JH, Kim JY, Carvalho AF, Seeman MV, Correll CU, Fusar-Poli P. Age at onset of mental disorders worldwide: large-scale meta-analysis of 192 epidemiological studies. Mol Psychiatry. 2022;27:281–95. 34079068 10.1038/s41380-021-01161-7 PMC8960395

[58] Visit to the. child welfare center, BVC – 1177. https://www.1177.se/Stockholm/barn--gravid/vard-och-stod-for-barn/besok-pa-barnavardscentralen-bvc/?gad_source=1&gad_campaignid=8632436095&gbraid=0AAAAADCu4kyrNqD9MVDnkjMKaVT7BELKB&gclid=CjwKCAjwqazPBhALEiwAOuXqdAPO7RqgOTkhdTmQwjhip4sd_so5rT5GA4Pn3oIpJWlDd8jwWIuehRoCECUQAvD_BwE

[59] László KD, Chen H, Andersson F, Galanti MR. Preschool care early in life and mental health in adolescence in Sweden: a cohort study. BMJ Open. 2026;16:e105111. 10.1136/BMJOPEN-2025-105111. 41997704 10.1136/bmjopen-2025-105111 PMC13110637

[60] National Child Health Care Program - National Handbook of Child Health Care. https://www.rikshandboken-bhv.se/verksamhetsriktlinjer/barnhalsovardens-nationella-program/

[61] BVC-ELVIS -. a new methodology at the 18-month visit at BVC | Karolinska Institutet. https://ki.se/forskning/forskningsomraden-centrum-och-natverk/forskargrupper/epidemiologi-och-interventioner-psykisk-halsa-substansbruk-och-social-kontext-emilie-agardhs-renee-gardners-forskargrupp/bvc-elvis-ett-nytt-18-manadersbesok-pa-barnavardscentralerna-i-region

[62] Föräldraskapsstödskartan. (mfof.se). https://www.mfof.se/foraldraskapsstod/program-och-metoder/foraldraskapsstodskartan.html

