# Supplementary material for: Healthcare utilization and costs of early childhood mental health problems: a longitudinal analysis using multi-rater assessments and Swedish national registers
Source: Child Adolesc Psychiatry Ment Health. 2026 Jun 12;20:84. doi: 10.1186/s13034-026-01110-8 (PMC13267661; doi:10.1186/s13034-026-01110-8)
Supplement: Supplementary file 1 — Supplementary Material 1. [file 13034_2026_1110_MOESM1_ESM.docx]

**Supplementary Online Resource**

# Healthcare utilization and costs of early childhood mental health problems: a longitudinal analysis using multi-rater assessments and Swedish national registers

**Authors:** Ingyin Moe^1^, Natalie Durbeej^1,2^, Claire de Oliveira ^3,4^, Filipa Sampaio^1,5^

**Affiliations**

^1^ Department of Public Health and Caring Sciences, Uppsala University, Sweden

^2^ Department of Health Sciences, Karlstad University, Sweden

^3^ Campbell Family Mental Health Research Institute and Institute for Mental Health Policy Research, Centre for Addiction and Mental Health, Toronto, Canada

^4^ Institute of Health Policy, Management and Evaluation, Dalla Lana School of Public Health, University of Toronto, Toronto, Canada

^5^ Department of Learning, Informatics, Management and Ethics, Karolinska Institutet, Sweden

**ORCID ID**

Ingyin Moe: 0009-0008-9951-9078

Natalie Durbeej: 0000-0001-9916-0087s

Claire de Oliveira: 0000-0003-3961-6008

Filipa Sampaio: 0000-0002-5540-9853

**Corresponding author**

Dr. Ingyin Moe

Department of Public Health and Caring Sciences

Uppsala University

Husargatan 3, 751 22 Uppsala, Sweden

[ingyin.moe@uu.se](mailto:ingyin.moe@uu.se)

+46(0)765669724

Table 1. Variables coding and reclassification

| **Variables** | **Level** | **Recode** |
| --- | --- | --- |
| Parental marital status | 1 = Single | 2+3 = 1, Married/cohabiting |
|  | 2 = Cohabiting partner | 1+4+5 = 2, Other |
|  | 3 = Married |  |
|  | 4 = In a relationship |  |
|  | 5 = Other |  |
| Parental education | 1 = Unfinished primary school | 1+2 = 1, Primary school |
|  | 2 = Primary school (completed) | 3 = 2, High school |
|  | 3 = High school | 4+5 = 3, University |
|  | 4 = University/college (less than 3 years) |  |
|  | 5 = University/college (3 years or more) |  |
| Number of children in the family | 1 | 1 child |
|  | 2 | 2 children |
|  | 3 | ≥ 3 children |
|  | 4 |  |
|  | 5 |  |
|  | 6 |  |
|  | 7 |  |
|  | 8 |  |
| Parental psychological distress | GHQ score 0 to 11 | No (With parental distress) |
|  | GHQ score ≥12 | Yes (without parental distress) |

## Yearly average cumulative healthcare utilization and costs (Mother-rated SDQ)

Yearly average cumulative healthcare utilization for children with and without early mental health problems is displayed in **Figure 1.** An overall upward trend in utilization can be seen from 2016 to 2021 despite the initial fluctuations in children with early mental health problems. Highest peak can be seen in total utilization with almost 35 total visits in year 2021 for children with early mental health problems while their healthy peers showed small fluctuations with a gradual downward trend in late 2020. Utilization of primary care mirrored the similar trend as total utilization in children with early mental health problems, with notable fluctuations between 2016-2019 followed by a consistent increase through 2021. Prescribed medication use exhibited a steady climb throughout the years in both groups, reaching the peak at 2020, and slightly declined afterwards until 2021. Specialized outpatient care showed a sharp decrease in utilization for children with early mental health problems from 2016 to 2017, followed by a steady increase in subsequent years. Meanwhile, children with no early mental health problems experience a steep climb in 2017 and then remained relatively stable thereafter. Inpatient care remained low and relatively constant across all years for both groups, indicating minimal use of this type of healthcare.

**
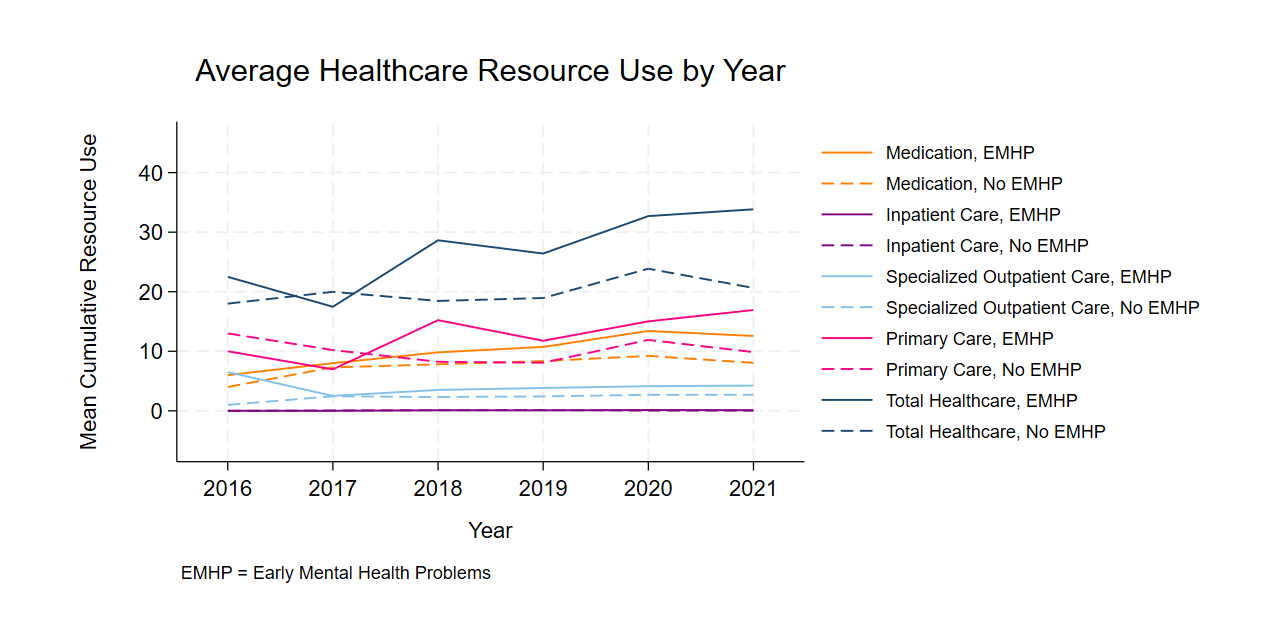
**

Legend: EMHP: Early mental health problems

Fig1. Yearly average healthcare utilization (Mother-rated SDQ)

**Figure 2** illustrates the yearly average cumulative healthcare costs for children with and without early mental health problems. From 2017 to 2021, children with early mental health problems experienced a consistent rise in total healthcare costs, with the highest average of approximately US$5,000 in 2021. In contrast, children without problems exhibited more variable cost patterns, with a modest decline beginning after 2020. Primary care and specialized outpatient care costs also increased steadily over time for the early mental health problems group. Meanwhile, these costs remained relatively stable for healthy children, showing only minor fluctuations. Prescribed medication costs for children with early mental health problems rose steadily until peaking in 2019, after which a slight decline occurred through 2021. A similar pattern, though at lower cost levels, was observed for children without early mental health problems. Inpatient care costs showed notable year-to-year variability in both groups. However, children with early mental health problems experienced a gradual increase in inpatient costs from 2019 to 2021, while costs remained relatively low and stable for their healthy peers.
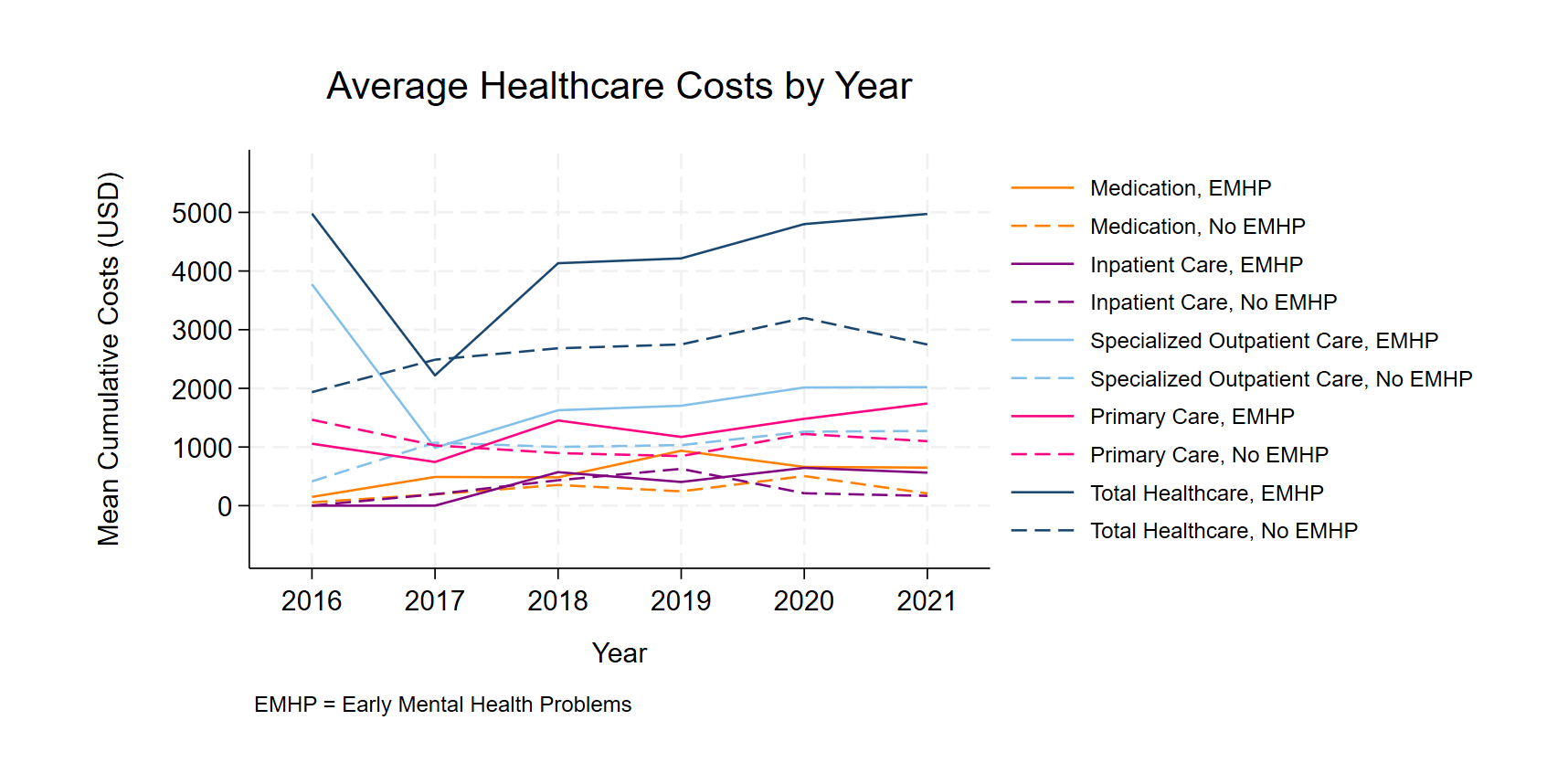


Legend: EMHP: Early mental health problems

Fig2. Yearly average healthcare costs (Mother-rated SDQ)

Table 2. Distribution and model fit check (Mother-rated SDQ)

| **Category** | **Overdispersion Test (Pearson χ²/df, p value) ^a^** | **Skeweness (stat, p value)** | **Heteroskedasticity (chi², p value) ^b^** | **Modified Park test ^c^** | **Zero fraction (%)** | **Model of choice** | **AIC** | **BIC** |
| --- | --- | --- | --- | --- | --- | --- | --- | --- |
| **Healthcare Utilization** |  |  |  |  |  |  |  |  |
| Primary care | 35.2, <0.001 | 6.28, <0.001 | -- | 2 | 19.40% | Hurdle model with negative binomial distribution | 8246.686 | 8329.635 |
| Inpatient care | 2.19, <0.001 | 8.26, <0.001 | -- | 2 | 94% | Hurdle model with negative binomial distribution | 658.78097 | 696.26074 |
| Outpatient care | 5.8, <0.001 | 3.21, <0.001 | -- | 2 | 27.11% | Hurdle model with negative binomial distribution | 658.78097 | 696.26074 |
| Medication | 34.2, <0.001 | 4.7, <0.001 | -- | 2 | 20.73% | Hurdle model with negative binomial distribution | 7542.5148 | 7625.1817 |
| Total healthcare | 43.46, <0.001 | 4.23, <0.001 | -- | 2 | 2.49% | GLM with negative binomial distribution | 10142.95 | 10168.43 |
| **Costs** |  |  |  |  |  |  |  |  |
| Primary care | -- | 5.36, <0.001 | 87.07, <0.001 | 2 | 19.40% | Two part model with gamma distribution | 17309.24 | 17360.19 |
| Inpatient care | -- | 7.0, <0.001 | 1.39, 0.238 | 2 | 94% | Two part model with gamma distribution | 1863.43 | 1914.381 |
| Outpatient care | -- | 3.9, <0.001 | 62.78, <0.001 | 2 | 27.11% | Two part model with gamma distribution | 16465.09 | 16516.04 |
| Medication | -- | 14.92, <0.001 | 151.32, <0.001 | 2 | 20.73% | Two part model with gamma distribution | 15295.12 | 15346.07 |
| Total healthcare | -- | 6.48, <0.001 | 42.77, <0.001 | 2 | 2.49% | GLM with gamma distribution | 22065.96 | 22091.44 |
|  |  |  |  |  |  |  |  |  |

^a^ Pearson χ²/df ratio > 1 indicates overdispersion

^b^ Breusch-Pagan Test p value < 0.05 → Reject null hypothesis of homoscedastic

^c^ Recommended family derived from coefficient for modified Park test: If coefficient ~=0, Gaussian, ~=1, Poisson, ~=2, Gamma/Negative binomial, ~=3, Inverse Gaussian or Wald [1]

Table 3. Missing covariates (Mother-rated SDQ)

| **Covariates** | | **N° Sample** | **Percentage** |
| --- | --- | --- | --- |
| **Sex** | |  |  |
|  | Girls | 3,318 | 49.38% |
|  | Boys | 3,402 | 50.62% |
|  | NA | - | - |
|  | Total | 6,720 | 100% |
| **Age** | |  |  |
|  | 3 years | 3,410 | 50.74% |
|  | 4 years | 1,814 | 26.99% |
|  | 5 years | 1,496 | 22.26% |
|  | NA | - | - |
|  | Total | 6,720 | 100% |
| **Maternal Level of Education** | |  |  |
|  | Primary School | 139 | 2.07% |
|  | High School | 1,545 | 22.99% |
|  | University | 4,872 | 72.50% |
|  | NA | 164 | 2.44% |
|  | Total | 6,720 | 100% |
| **Maternal Country of birth** | |  |  |
|  | Sweden | 5,647 | 84.03% |
|  | Other | 976 | 14.52% |
|  | NA | 97 | 1.44% |
|  | Total | 6,720 | 100% |
| **Maternal marital status** | |  |  |
|  | Married/Co-habiting | 6,347 | 94.45% |
|  | Other | 261 | 3.88% |
|  | NA | 112 | 1.67% |
|  | Total | 6,720 | 100% |
| **Number of children in the family** | |  |  |
|  | 1 | 1,200 | 17.86% |
|  | 2 | 4,033 | 60.01% |
|  | 3+ | 1,366 | 20.33% |
|  | NA | 121 | 1.80% |
|  | Total | 6,720 | 100% |
| **Maternal mental health** | |  |  |
|  | Psychological distress | 1,603 | 23.85% |
|  | No psychological distress | 5,024 | 74.76% |
|  | NA | 93 | 1.38% |
|  | Total | 6,720 | 100% |


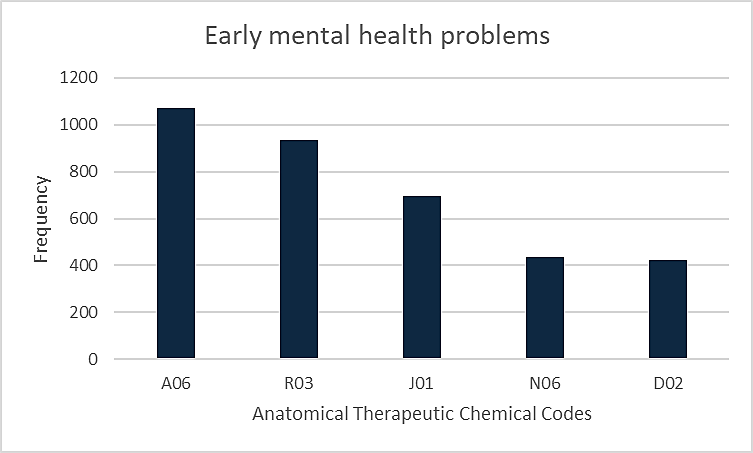

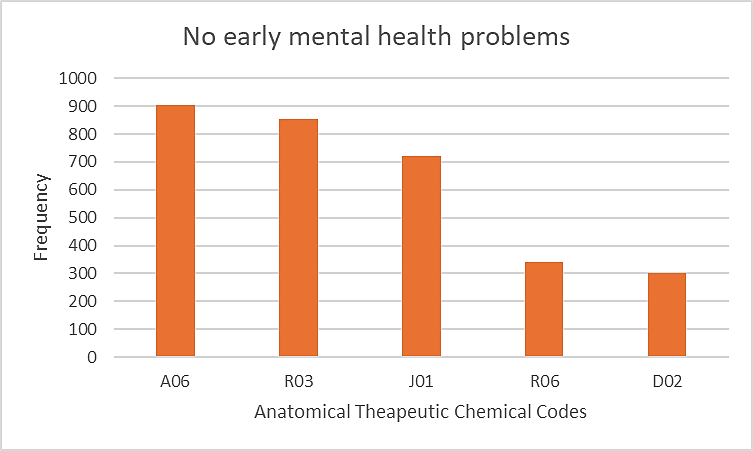


Fig 3. Top five commonly prescribed medications based on anatomical therapeutic chemical codes in children with and without early mental health problems

*Legend:*

*A06: Drugs for Constipation*

*R03: Drugs for Obstructive Airway Diseases (Asthma)*

*J01: Antibacterials for Systemic Use*

*N06: Psychoanaleptics (ADHD medications + antidepressants)*

*R06: Antihistamines for Systemic Use*

*D02: Emollients and Protectives*

*
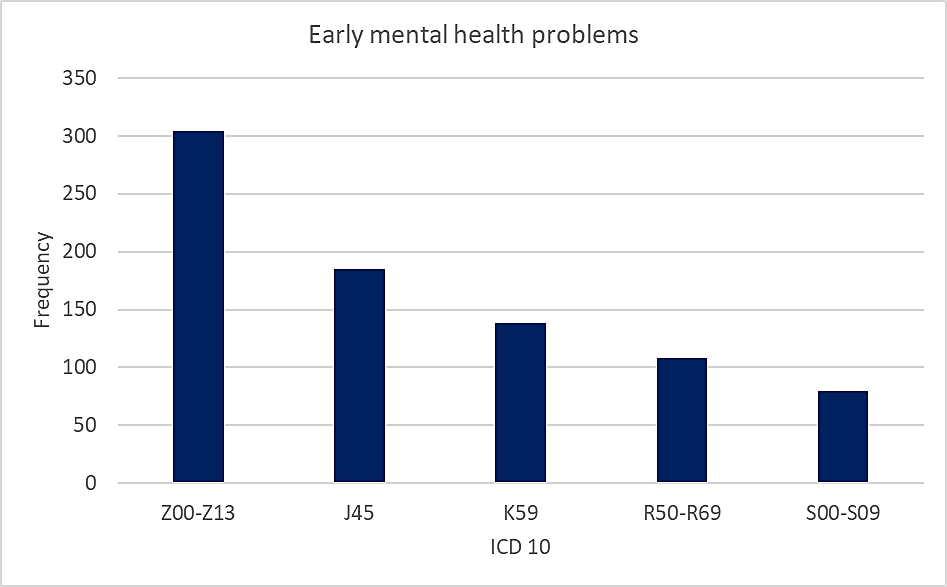
* *
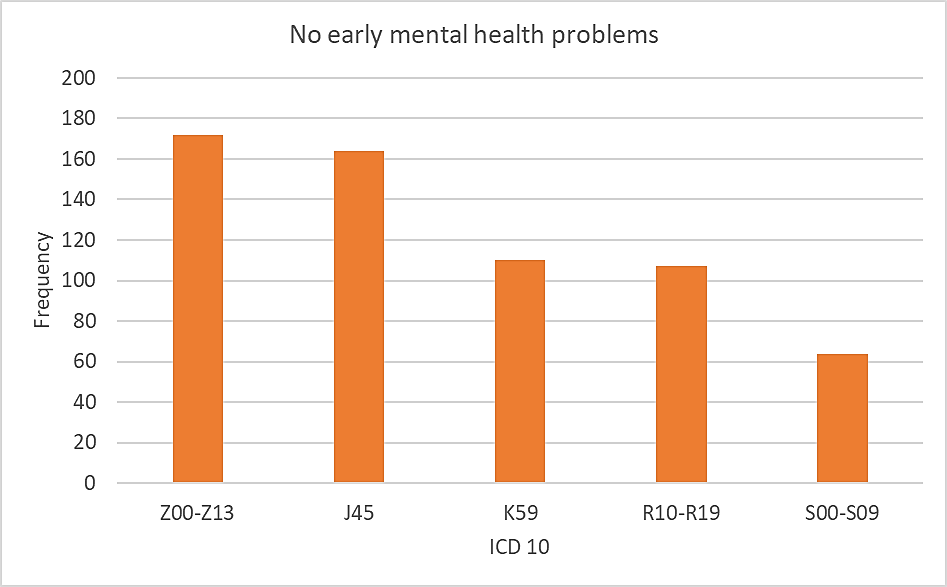
*

Fig 4. Top five non-psychiatric related ICD-10 diagnoses in children with and without early mental health problems

*Legend:*

*Z00–Z13: Persons encountering health services for examination and investigation*

*J45: Asthma*

*K59: Other functional intestinal disorders*

*R50–R69: General symptoms and signs*

*S00–S09: Injuries to the head*


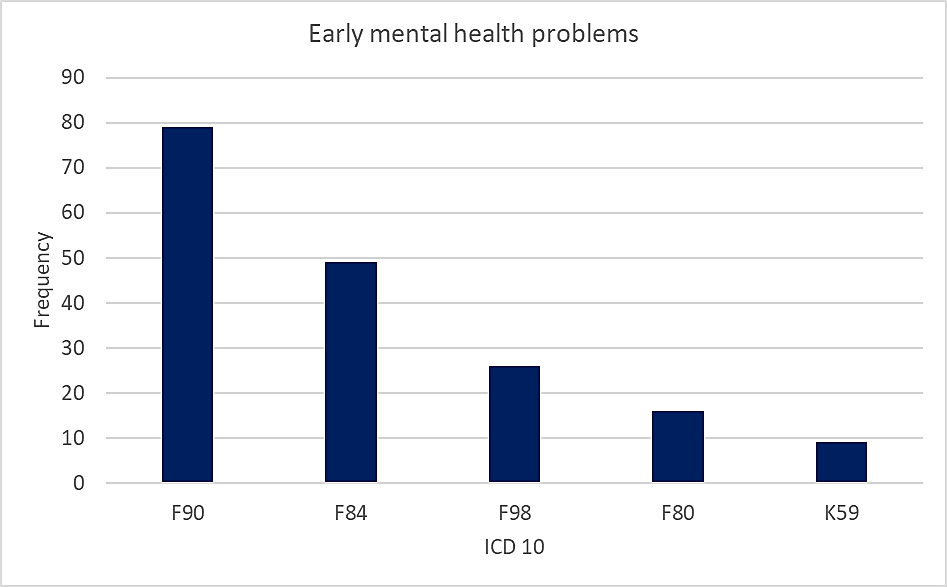

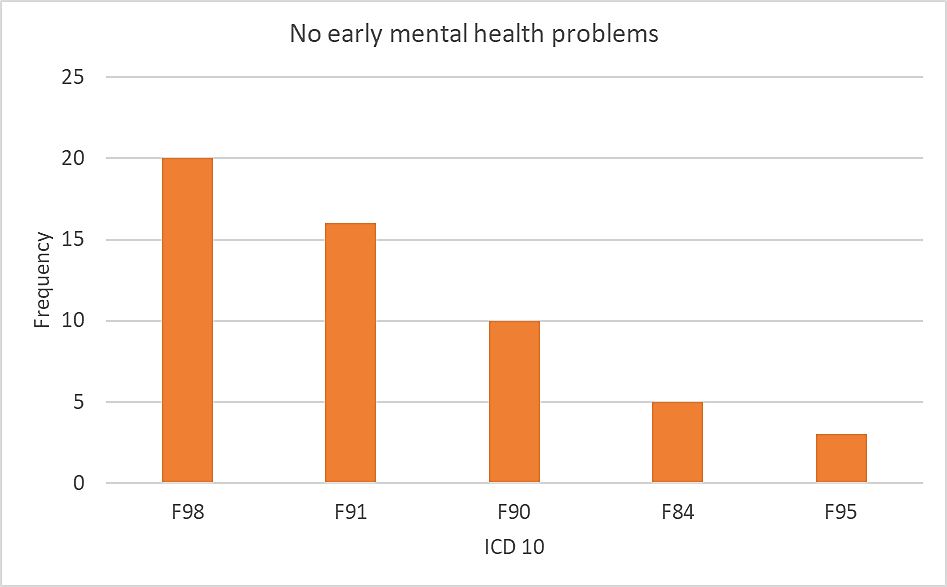


Fig 5. Top five psychiatric-related ICD-10 diagnoses in children with and without early mental health problems

*Legend:*

*F98: Other behavioural and emotional disorders with onset usually occurring in childhood and adolescence*

*F91: Conduct disorders*

*F90: Hyperkinetic disorders*

*F84: Pervasive developmental disorders*

*F95: Tic disorders*

*F80: Specific developmental disorders of speech and language*

*K59: Other functional intestinal disorders*

Table 4. Mean healthcare utilizations and costs for children with early mental health problems by sex (Mother-rated SDQ)

| **Health Service Category** | | **Total (n=603)** | **Girls (n=298)** | **Boys (n=305)** | **Between-group difference** |
| --- | --- | --- | --- | --- | --- |
| **Prescribed Medication** | |  |  |  |  |
| **Utilization** | |  |  |  |  |
|  | n | 493 | 243 | 250 |  |
|  | Mean (SE) | 11.09 (0.80) | 10.41 (1.09) | 11.75 (1.19) | -1.34 (1.61) |
|  | Median (IQR) | 3 (1-11) | 3 (1-11) | 4 (1-12) |  |
|  | 95%CI | 9.50, 12.68 | 8.26, 12.56 | 9.41, 14.09 | -4.50, 1.82 |
| **Costs (in USD) ^a^** | |  |  |  |  |
|  | Mean (SE) | 624 (131) | 673 (241) | 577 (111) | 96 (265) |
|  | Median (IQR) | 67 (18-282) | 62 (16-271) | 75 (19-287) |  |
|  | 95%CI | 366, 882 | 198, 1147 | 359, 795 | -424, 616 |
| **Inpatient Care** | |  |  |  |  |
| **Utilization** | |  |  |  |  |
|  | n | 41 | 23 | 18 |  |
|  | Mean (SE) | 0.09 (0.02) | 0.10 (0.02) | 0.10 (0.03) | 0 (0.04) |
|  | Median (IQR) | 0 (0-0) | 0 (0-0) | 0 (0-0) |  |
|  | 95%CI | 0.06, 0.13 | 0.06, 0.14 | 0.04, 0.14 | -0.071, 0.071 |
| **Costs (in USD) ^a^** | |  |  |  |  |
|  | Mean (SE) | 506 (91) | 552 (132) | 462 (125) | 90 (182) |
|  | Median (IQR) | 0 (0-0) | 0 (0-0) | 0 (0-0) |  |
|  | 95%CI | 328, 685 | 292, 811 | 216, 708 | -266, 446 |
| **Specialized Outpatient Care** | |  |  |  |  |
| **Utilization** | |  |  |  |  |
|  | n | 474 | 222 | 252 |  |
|  | Mean (SE) | 3.74 (0.20) | 3.32 (0.26) | 4.14 (0.29) | -0.82 (0.39) |
|  | Median (IQR) | 2 (1-5) | 2 (0-4) | 3 (1-5) |  |
|  | 95%CI | 3.35, 4.12 | 2.80, 3.84 | 3.58, 4.71 | -1.58, -0.06 |
| **Costs (in USD) ^a^** | |  |  |  |  |
|  | Mean (SE) | 1,739 (103) | 1,566 (148) | 1,909 (142) | -343 (205) |
|  | Median (IQR) | 888 (357-2,133) | 753 (0-1,972) | 1171 (390-2,341) |  |
|  | 95%CI | 1,538, 1941 | 1274, 1,857 | 1,630, 2,188 | -745, 59 |
| **Primary Care** | |  |  |  |  |
| **Utilization** | |  |  |  |  |
|  | n | 488 | 232 | 256 |  |
|  | Mean (SE) | 14.04 (1.05) | 11.43 (0.91) | 16.59 (1.87) | -5.16 (2.08) |
|  | Median (IQR) | 8 (2-16) | 7 (1-15) | 8 (2-16) |  |
|  | 95%CI | 11.98, 16.10 | 9.64, 13.22 | 12.92, 20.27 | -9.24, -1.09 |
| **Costs (in USD) ^a^** | |  |  |  |  |
|  | Mean (SE) | 1,385 (90) | 1,174 (81) | 1,590 (158) | -416 (178) |
|  | Median (IQR) | 777 (179-1,704) | 782 (135-1,646) | 777 (186-1,741) |  |
|  | 95%CI | 1,209, 1560 | 1,015, 1,334 | 1,280, 1,901 | -764, -68 |
| **Total Healthcare** | |  |  |  |  |
| **Utilization** | |  |  |  |  |
|  | n | 592 | 290 | 302 |  |
|  | Mean (SE) | 28.96 (1.55) | 25.26 (1.75) | 32.58 (2.53) | -7.32 (3.08) |
|  | Median (IQR) | 16 (8-34) | 14 (7-31) | 18 (9-37) |  |
|  | 95%CI | 25.92, 32.00 | 21.82, 28.69 | 27.60, 37.56 | -13.35, -1.29 |
| **Costs (in USD) ^a^** | |  |  |  |  |
|  | Mean (SE) | 4,255 (282) | 3,964 (441) | 4,539 (353) | -575 (565) |
|  | Median (IQR) | 2,127 (945-4,588) | 1,851 (780-4,455) | 2,389 (1,209-5,039) |  |
|  | 95%CI | 3,701, 4,808 | 3,097, 4,832 | 3,843, 5,234 | -1,682, 532 |
|  |  |  |  |  |  |
| ^a^ Costs in 2024 USD | |  |  |  |  |
| Legend: SE: Standard Error; IQR: Interquartile range; CI: Confidence interval | | | | |  |

Table 5. Mean healthcare utilization and costs for children with early mental health problems by sex and symptom profiles (Mother-rated SDQ)

| **Health Service Category** | |  | **Externalizing problems** | | **Internalizing problems** | | **Both externalizing and internalizing problems** | |
| --- | --- | --- | --- | --- | --- | --- | --- | --- |
|  |  | **Total (n=603)** | **Boys (n=128)** | **Girls (n=124)** | **Boys (n=73)** | **Girls (n=98)** | **Boys (n=104)** | **Girls (n=69)** |
| **Prescirbed Medication** | |  |  |  |  |  |  |  |
| **Utilization** | |  |  |  |  |  |  |  |
|  | n | 493 | 106 | 99 | 60 | 83 | 84 | 57 |
|  | Mean (SE) | 11.09 (0.80) | 13.41 (2.10) | 9.52 (1.64) | 12.68 (2.49) | 9.10 (1.30) | 9.05 (1.55) | 14.04 (3.17) |
|  | Median (IQR) | 3 (1-11) | 3 (1-13) | 3 (1-8.5) | 5 (1-15) | 3 (1-11) | 4 (1-10) | 4 (1-15) |
|  | 95%CI | 9.50, 12.68 | 9.25, 17.56 | 6.27, 12.76 | 7.71, 17.65 | 6.53, 11.67 | 5.98, 12.12 | 7.72, 20.37 |
| **Costs (in USD) ^a^** | |  |  |  |  |  |  |  |
|  | Mean (SE) | 624 (131) | 823 (237) | 392 (103) | 565 (173) | 647 (396) | 283 (68) | 1,259 (858) |
|  | Median (IQR) | 67 (18-282) | 65 (21-313) | 47 (12-165) | 91 (19-354) | 67 (21-269) | 80 (17-216) | 93 (17-388) |
|  | 95%CI | 366, 882 | 354, 1,291 | 189, 596 | 220, 909 | -138, 1,432 | 148, 419 | -454, 2,971 |
| **Inpatient Care** | |  |  |  |  |  |  |  |
| **Utilization** |  |  |  |  |  |  |  |  |
|  | n | 41 | 6 | 11 | 2 | 7 | 10 | 5 |
|  | Mean (SE) | 0.09 (0.02) | 0.09 (0.05) | 0.11 (0.04) | 0.05 (0.04) | 0.09 (0.04) | 0.13 (0.04) | 0.09 (0.04) |
|  | Median (IQR) | 0 (0-0) | 0 | 0 (0-0) | 0 (0-0) | 0 (0-0) | 0 (0-0) | 0 (0-0) |
|  | 95%CI | 0.06, 0.13 | -0.01, 0.18 | 0.04, 0.19 | -0.03, 0.14 | 0.02, 0.16 | 0.04, 0.20 | 0.007, 0.17 |
| **Costs (in USD) ^a^** | |  |  |  |  |  |  |  |
|  | Mean (SE) | 506 (91) | 385 (190) | 696 (236) | 419 (297) | 556 (244) | 588 (192) | 342 (157) |
|  | Median (IQR) | 0 (0-0) | 0 | 0 (0-0) | 0 (0-0) | 0 (0-0) | 0 (0-0) | 0 (0-0) |
|  | 95%CI | 328, 685 | 9, 760 | 228, 1,163 | -173, 1,011 | 73, 1039 | 207, 970 | 29, 656 |
| **Specialized Outpatient Care** | |  |  |  |  |  |  |  |
| **Utilization** |  |  |  |  |  |  |  |  |
|  | n | 474 | 100 | 88 | 61 | 79 | 91 | 52 |
|  | Mean (SE) | 3.74 (0.20) | 3.66 (0.42) | 2.80 (0.33) | 4.05 (0.54) | 3.79 (0.56) | 4.80 (0.53) | 3.81 (0.56) |
|  | Median (IQR) | 2 (1-5) | 2 (1-5) | 2 (0-4) | 2 (1-5) | 2 (1-4) | 3.5 (2-6) | 2 (1-5) |
|  | 95%CI | 3.35, 4.12 | 2.83, 4.50 | 2.14, 3.45 | 2.97, 5.14 | 2.68, 4.89 | 3.74, 5.86 | 2.69, 4.94 |
| **Costs (in USD) ^a^** | |  |  |  |  |  |  |  |
|  | Mean (SE) | 1,739 (103) | 1,642 (202) | 1,333 (170) | 1,935 (308) | 1,825 (344) | 2,219 (253) | 1,729 (274) |
|  | Median (IQR) | 888 (357-2,133) | 870 (357-2,173) | 765 (0-1,599) | 888 (359-2,310) | 736 (339-2,063) | 1,543 (734-2,757) | 848 (262-2,382) |
|  | 95%CI | 1,538, 1,941 | 1,242, 2,042 | 996, 1,670 | 1,322, 2,548 | 1,143, 2,507 | 1,718, 2,721 | 1,183, 2276 |
| **Primary Care** | |  |  |  |  |  |  |  |
| **Utilization** |  |  |  |  |  |  |  |  |
|  | n | 488 | 112 | 99 | 73 | 73 | 82 | 55 |
|  | Mean (SE) | 14.04 (1.05) | 11.91 (1.50) | 10.27 (0.90) | 21.04 (5.59) | 10.17 (1.17) | 19.23 (3.31) | 15.70 (3.12) |
|  | Median (IQR) | 8 (2-16) | 7 (2-17) | 8 (2-15) | 9 (2-14) | 7 (0-14) | 8 (1.5-17) | 9 (2-16) |
|  | 95%CI | 11.98, 16.10 | 8.95, 14.87 | 8.50, 12.05 | 9.89, 32.19 | 7.84, 12.50 | 12.66, 25.80 | 9.46, 21.93 |
| **Costs (in USD) ^a^** | |  |  |  |  |  |  |  |
|  | Mean (SE) | 1,385 (90) | 1,240 (147) | 1,081 (94) | 1,821 (423) | 1,083 (125) | 1,860 (304) | 1,513 (244) |
|  | Median (IQR) | 777 (179-1,704) | 739 (183-1,707) | 805 (179-1,630) | 876 (268-1,533) | 675 (0-1,432) | 766 (133-1,792) | 899 (179-1,859) |
|  | 95%CI | 1,209, 1,560 | 949, 1,531 | 895, 1268 | 978, 2,663 | 835, 1,330 | 1,257, 2,463 | 1,025, 2,000 |
| **Total Healthcare** | |  |  |  |  |  |  |  |
| **Utilization** |  |  |  |  |  |  |  |  |
|  | n | 592 | 126 | 120 | 72 | 95 | 104 | 69 |
|  | Mean (SE) | 28.96 (1.55) | 29.07 (3.19) | 22.70 (2.46) | 37.84 (6.99) | 23.15 (2.24) | 33.20 (3.95) | 33.64, 5.09 |
|  | Median (IQR) | 16 (8-34) | 18 (8-34.5) | 12 (6.5-29) | 19 (11-35) | 13 (8-31) | 17.5 (10-38.5) | 16 (7-36) |
|  | 95%CI | 25.92, 32.00 | 22.74, 35.40 | 17.84, 27.57 | 23.90, 51.77 | 18.71, 27.59 | 25.38, 41.03 | 23.49, 43.79 |
| **Costs (in USD) ^a^** | |  |  |  |  |  |  |  |
|  | Mean (SE) | 4,255 (282) | 4,089 (549) | 3,503 (429) | 4,740 (816) | 4,111 (922) | 4,951 (541) | 4,843 (1147) |
|  | Median (IQR) | 2,127 (945-4,588) | 2157 (1,034-4,517) | 1,544 (745-4,298) | 2,389 (1,121-3,886) | 2,014 (848-3,683) | 2,711 (1,571-6,442) | 2,595 (950-4,870) |
|  | 95%CI | 3,701, 4,808 | 3,002, 5,175 | 2,652, 4,353 | 3,114, 6,366 | 2,282, 5,940 | 3878, 6025 | 2,555, 7,131 |
|  |  |  |  |  |  |  |  |  |

^a^ Costs in 2024 USD

Legend: SE: Standard error; IQR: Interquartile range; CI: Confidence interval

Table 6. Incremental healthcare utilization and associated costs of children with early mental health problems (Father-rated SDQ)

| **Children with early mental health problems (n=801)** | **Marginal effects (95%CI)** | |
| --- | --- | --- |
| **Prescribed Medication** |  |  |
| Utilization | 3.16 (1.49, 4.83)*** | |
| Costs (in USD) ^a^ | 331 (77, 584)* | |
| **Inpatient Care** |  |  |
| Utilization | 0.05 ( -0.006, 0.10) | |
| Costs (in USD) ^a^ | 288 (10, 565)* | |
| **Specialized Outpatient Care** |  |  |
| Utilization | 1.06 (0.59, 1.53)*** | |
| Costs (in USD) ^a^ | 563 (313, 813)*** | |
| **Primary Care** |  |  |
| Utilization | 3.57 (1.91, 5.23)*** | |
| Costs (in USD) ^a^ | 323 (172, 473)*** | |
| **Total Healthcare** |  |  |
| Utilization | 7.91 (5.65, 10.18)*** | |
| Costs (in USD) ^a^ | 1,563 (912, 2,213)*** | |
|  |  |  |
| ***: Statistically significant at pvalue 0.001 | | |
| *: Statistically significant at pvalue 0.05 | | |
| CI: Confidence Interval |  |  |
| ^a^ Costs in 2024 USD |  |  |

Table 7. Incremental healthcare utilization and associated costs of children with early mental health problems (Teacher-rated SDQ)

| **Children with early mental health problems (n=535)** | **Marginal effects (95%CI)** | | |
| --- | --- | --- | --- |
| **Prescribed Medication** |  |  |  |
| Utilization | 2.55 (-0.20, 5.30) | |  |
| Costs (in USD) ^a^ | 268 (14, 522)* | |  |
| **Inpatient Care** |  |  |  |
| Utilization | 0.02 (-0.02, 0.07) | |  |
| Costs (in USD) ^a^ | 137 (-94, 367) | |  |
| **Specialized Outpatient Care** |  | |  |
| Utilization | 0.89 (0.23, 1.54) * | |  |
| Costs (in USD) ^a^ | 448 (124, 771)* | |  |
| **Primary Care** |  | |  |
| Utilization | 3.26 (1.09, 5.42)* | |  |
| Costs (in USD) ^a^ | 272 (71, 473)* | |  |
| **Total Healthcare** |  | |  |
| Utilization | 6.32 (3.17, 9.48)*** | |  |
| Costs (in USD) ^a^ | 1,255 (524, 1,986)* | |  |
|  |  |  |  |
| ***: Statistically significant at pvalue 0.001 | |  |  |
| *: Statistically significant at pvalue 0.05 | |  |  |
| CI: Confidence Interval |  |  |  |
| ^a^ Costs in 2024 USD |  |  |  |

## Population level estimates

Additionally, population-level cost estimates were calculated using a prevalence of 6.7% screening positive for early mental health problems among pre-school children based on a study undertaken in Norway [2]. The choice for the prevalence value was based on two reasons: 1) similar age group and country context as both Norway and Sweden are Nordic countries, and 2) the study used SDQ as a screening tool for mental health problems in children, which is a similar approach undertaken in our study. In order to calculate the population-level prevalence, 2024 population level data was retrieved from Statistics Sweden (SCB: Statistikdatabasen) [3], and then multiplied with the prevalence value. The result was multiplied again with the average excess cost for each health service category, and then presented as a proportion of 2024 Swedish national healthcare budget retrieved from the Government Offices of Sweden [4].

Table 8. Population-level cost estimates and comparison with 2024 Sweden national healthcare budget

| Health services | Prevalence ^a^ | Population aged 3-5 years old in Sweden ^b^ | Estimated population with EMHP | Excess cost (USD) ^b^ | Excess cost (SEK) ^b^ | National healthcare budget in USD (SEK) ^b c^ | Proportion of national healthcare budget (%) ^b^ |
| --- | --- | --- | --- | --- | --- | --- | --- |
| Prescribed Medication | 0.067 | 350,719 | 23,499 | 7,025,954 | 62,074,304 | 13.13 (116) billion | 0.05% |
| Inpatient Care |  |  |  | 4,112,180 | 36,331,110 |  | 0.03% |
| Specialized Outpatient Care |  |  |  | 14,615,864 | 129,131,159 |  | 0.11% |
| Primary Care |  |  |  | 8,835,313 | 78,059,991 |  | 0.07% |
| Total healthcare |  |  |  | 34,236,838 | 302,482,465 |  | 0.26% |

^a^ Using prevalence of early mental health problems in preschool children in Norway [2]

^b^ 2024 data

^c^ Values in billion

Legend: EMHP: Early Mental Health Problems; SEK: Swedish Kronor

**Reference**

1. Deb, P., Norton, E.C.: Modeling Health Care Expenditures and Use. Annu. Rev. Public Health. 39, 489–505 (2018). https://doi.org/10.1146/ANNUREV-PUBLHEALTH-040617-013517/CITE/REFWORKS

2. Sveen, T.H., Berg-Nielsen, T.S., Lydersen, S., Wichstrøm, L.: Detecting Psychiatric Disorders in Preschoolers: Screening With the Strengths and Difficulties Questionnaire. J. Am. Acad. Child Adolesc. Psychiatry. 52, 728–736 (2013). https://doi.org/10.1016/J.JAAC.2013.04.010

3. Folkmängden efter region, civilstånd, ålder och kön. År 1968 - 2024. PxWeb, https://www.statistikdatabasen.scb.se/pxweb/sv/ssd/START__BE__BE0101__BE0101A/BefolkningNy/?loadedQueryId=129324&timeType=top&timeValue=1

4. Regeringskansliet, R. och: Central government budget in figures. (2019)
